# Plant functional group has stronger effects on soil functions than planting density: an examination with pot experiment

**DOI:** 10.3389/fpls.2025.1652236

**Published:** 2025-09-22

**Authors:** Huan Jiao, Zhenrui Cao, Lin Gong, Xingchen Chang, Chuanxu Fang, Xiang Wang, Wenrao Li, Satoshi Ishii, Weibo Kong, Xiaorong Wei

**Affiliations:** ^1^ College of Natural Resources and Environment, Northwest A&F University, Yangling, Shaanxi, China; ^2^ College of Soil and Water Conservation Science and Engineering, Northwest A&F University, Yangling, Shaanxi, China; ^3^ State Key Laboratory of Soil and Water Conservation and Desertification Control, Northwest A&F University, Yangling, Shaanxi, China; ^4^ College of Land Science and Technology, China Agricultural University, Beijing, China; ^5^ School of Life Sciences, Henan University, Kaifeng, Henan, China; ^6^ Department of Soil, Water, and Climate, University of Minnesota, St. Paul, MN, United States; ^7^ BioTechnology Institute, University of Minnesota, St. Paul, MN, United States

**Keywords:** planting density, plant functional groups, soil enzyme activities, soil microbial necromass carbon, soil nitrogen mineralization, soil multifunctionality

## Abstract

**Introduction:**

Plant functional groups (PFGs) and plant density are two important characteristics of plant community dynamics; however, the main and interactive effects of PFGs and plant density on ecosystem functions have not been thoroughly analyzed, limiting our ability to predict and manage ecological responses to community changes.

**Methods:**

A pot experiment was done with four PFGs (C_3_ grasses, C_4_ grasses, forbs, and legumes) and six planting density gradients (1, 2, 4, 8, 12, and 16 spots per pot) to evaluate how PFGs and plant densities influence plant biomass and soil functions, including nutrients, extracellular enzyme activity, microbial necromass carbon, and nitrogen mineralization rates, in rhizosphere and bulk soils.

**Results:**

Most of the soil function metrics increased as planting density increased, and such effects were greater in rhizosphere soils than in bulk soils. The magnitude and direction of density effects varied among PFGs, indicating interactive effects. Legumes had stronger effects than the other PFGs on soil multifunctionality index, nitrogen mineralization rates, and aboveground biomass. Similarly, C_3_ grasses had the strongest effects on soil extracellular enzyme activities in rhizosphere soils among the four PFGs tested.

**Conclusion:**

Our results suggest that plant functional group has stronger effects on soil functions than planting density.

## 1 Introduction

Plant density and plant functional groups (PFGs) are important characteristics of plant communities. PFGs are composed of species that share morphological, physiological, and phenological traits (Calbi et al., 2024). Both factors influence ecosystem functions, stability, and services, and are often affected by natural (e.g., wildfires and droughts, Oñatibia et al., 2020) or anthropogenic disturbances (e.g., overgrazing, tillage and vegetation rehabilitation, Nie and Zollinger, 2012; Dai et al., 2023). Changes in plant density and PFGs can influence plant population structure and inter- and intra-species competitions (Yu et al., 2019), resulting in alterations in ecosystem functions. It is well known that plant communities influence ecosystem processes (Wei et al., 2019; Grau-Andres et al., 2020). However, the impact of plant density and PFGs—important components of plant communities—on ecosystem processes such as biogeochemical cycling has not been thoroughly investigated. Clarifying both their main and interactive effects could advance our understanding of how plant community change influences ecological processes and enhance our ability to manage plant ecosystems more effectively.

Plant interactions involve a dynamic balance between competition and facilitation. While plants compete for essential resources such as light, nutrients, water, space, and pollinators, they may also facilitate each other by reducing herbivory, alleviating abiotic stress, and enhancing resource availability. Such facilitation can occur through various mechanisms, including canopy leachates, microbial stimulation, and mycorrhizal networks (Postma et al., 2020). These contrasting relationships often occur simultaneously and are strongly influenced by plant density (Zhang and Tielborger, 2020a). Density determines the intensity of interspecific competition and directly affects plant growth, reproduction, and ultimately soil properties and ecosystem functions (Han et al., 2008). Within an optimal range, increasing plant density can enhance resource acquisition, vegetation productivity, and ecosystem carbon (C) storage (Zhang et al., 2024). However, excessive density may lead to reduced individual performance and reproductive investment, potentially destabilizing food webs and impairing ecosystem resilience (Marquard et al., 2009). Notably, the positive relationship between biodiversity and productivity may be largely mediated by density-dependent effects rather than community size (Marquard et al., 2009). Currently, most existing studies on plant density have focused on productivity, especially in forest and agricultural ecosystems (Du et al., 2024; Li et al., 2024). The impacts of density variation on various soil functions in grasslands—such as soil nitrogen (N) mineralization, extracellular enzyme activities, and microbial necromass C—have yet to be systematically investigated.

Differences among PFGs are key determinants of ecosystem functions, as they often better explain ecosystem processes than species identity by capturing variation in traits related to productivity, nutrient cycling, and stress tolerance (Yan et al., 2020). In grassland ecosystems, plant species are commonly classified into legumes, non-leguminous forbs, C_3_ grasses, and C_4_ grasses based on their functional roles in productivity (Mitchell et al., 2003; Gui et al., 2018; Yang et al., 2019). Legumes, in particular, enhance soil N availability, primary productivity, and C sequestration due to their N-fixing capacity, thereby improving ecosystem resistance to disturbance (Gao et al., 2017). Meanwhile, C_4_ grasses are often associated with higher biomass accumulation, owing to their more efficient photosynthesis, greater stress tolerance, and superior resource-use efficiency (Somerville et al., 2010). Comparing how distinct PFGs modulate productivity and key ecosystem processes (e.g., nutrient cycling) could provide a mechanistic basis for optimizing land management.

Soil N mineralization, which is driven by soil microorganisms, determines the availability of N for plant growth. Soil N mineralization can also be affected by the quality and quantity of plant litter, soil organic matter (SOM), root exudates, and root distribution (Chapman et al., 2005; Gan et al., 2021). Plant fine roots can be quickly degraded and release N into soils, thereby accelerating net soil N mineralization rates (Fornara et al., 2009). Similarly, PFGs can influence microbial activities related to soil nutrient cycling (Gastine et al., 2003; Sun et al., 2020). For example, legumes can increase microbial biomass and reduce microbial necromass N reuse through symbiotic N fixation, thereby increasing the accumulation of microbial necromass C and soil organic carbon (SOC) content (Jia et al., 2022). However, it remains unclear how soil functions vary with plant density and PFGs.

The rhizosphere is the area most strongly affected by plant roots and is sensitive to plant community change (Yue et al., 2023). Plant roots release various organic compounds (e.g., organic acids, carbohydrates, amino acids) into the surrounding soils, influencing the microbial and biogeochemical characteristics of rhizosphere soils (Huo et al., 2022). It has been shown that the soil ecological processes differed significantly between bulk and rhizosphere soils (Wang et al., 2022; Han et al., 2024). For instance, soil bacterial diversity as well as the soil enzyme activities related to C, N, and phosphorus (P) cycling were higher in the rhizosphere soils of *Robinia pseudoacacia* L. than in bulk soils (Yang et al., 2017). Similarly, the gross N mineralization rates in the rhizosphere soils of *Avena barbata* were about ten times faster than those in bulk soils, even though the nitrification potential was similar between the two soils (Herman et al., 2006). However, it is unclear how soil functions in rhizosphere and bulk soils are affected by changes in plant density and PFGs.

Previous studies suggest that PFGs differ in their effects on soil functions. In particular, legumes may have a more pronounced positive effect due to their symbiotic associations with nitrogen-fixing microbes, which increase nitrogen availability and benefit soil microbial communities (Gou et al., 2023). In addition, planting density has been shown to positively saturate plant biomass accumulation (Stachova et al., 2013), and increased biomass can directly enhance soil functions through mechanisms such as root exudation and microbial activation (Gilmullina et al., 2023). Furthermore, the impact of planting density on soil functions may vary among functional groups, as plant–soil feedbacks are often group-specific and can differentially influence microbial activity and nutrient cycling (Mariotte et al., 2018). Based on these considerations, we hypothesized that (H1) legumes exert a more pronounced positive effect on soil functions compared to other PFGs; (H2) soil functions exhibit nonlinear responses to increasing planting density; (H3) the effects of planting density on soil functions differ significantly among PFGs. To test these hypotheses, we did a pot experiment to examine the effects of four functional groups (C_3_ grasses, C_4_ grasses, forbs, and legumes) and six planting densities (1, 2, 4, 8, 12, 16 spots per pot) on soil functions in both bulk and rhizosphere soils.

## 2 Materials and methods

### 2.1 Study site and soils

This study was designed to investigate the effects of PFGs and planting density on soil functions using a pot experiment. The experiment was conducted from March to September 2021 at Northwest A&F University, Yangling, Shaanxi, China (34°16′49″ N, 108°4′59″ E). The study site has a warm temperate semi-humid continental climate, with a mean annual temperature of 12.9°C and mean annual precipitation of 649.5mm, respectively. To minimize the influence of anthropogenic disturbances and legacy effects from surface management, the soil used for the pot experiment was collected from a depth of 1.5–2.5 m in grassland in Yangling, Shaanxi Province, China in February 2021. This subsoil was selected to reduce background biological activity, residual fertilizers, and microbial legacy from topsoil, thereby enabling a more controlled assessment of soil functions under varying plant densities and functional group treatments. The soil is classified as Anthrosol according to the Food and Agriculture Organization classification (FAO and ITPS, 2015), with a texture of silty clay loam. The soil was air-dried, passed through an 8mm sieve and homogenized for the pot experiment. The soil has a pH of 8.46, SOC of 4.46g kg^-1^, total nitrogen (TN) of 0.72g kg^-1^, ammonium (NH_4_^+^) of 1.02 mg kg^-1^, nitrate (NO_3_^–^) of 10.73 mg kg^-1^, total phosphorus (TP) of 0.75g kg^-1^, and available phosphorus (OP) of 7.69 mg kg^-1^.

### 2.2 Experimental design

The experiment involved 16 plant species classified into four PFGs based on their ecological functions (C_3_ grasses, C_4_ grasses, forbs, and legumes), with six planting density levels of 1, 2, 4, 8, 12, and 16 spots per pot (see **Supplementary Figure S1** for a schematic of the experimental design). Within each PFG, four representative plant species were selected to capture natural variation within the group. However, species identity was not treated as an independent treatment factor. Each species was grown in monoculture, and each species × density combination constituted an independent experimental unit with two biological replicates (n = 2). The results were interpreted at the functional group level, with species identity considered a source of variability rather than replication, thereby avoiding pseudo-replication and ensuring robust inference. The C_3_ grasses were *Leymus chinensis* (Trin. ex Bunge) Tzvelev, *Lolium perenne* L., *Bromus inermis* Leyss., and *Elymus dahuricus* Turcz. The C_4_ grasses were *Panicum virgatum* L., *Pennisetum alopecuroides* (L.) Spreng., *Setaria viridis* (L.) P. Beauv., and *Eragrostis pilosa* (L.) P. Beauv. The forbs were *Bidens biternata* (Lour.) Merr. & Sherff, *Bidens pilosa* L., *Ambrosia artemisiifolia* L., and *Ambrosia artemisiifolia* L. The legumes included *Medicago sativa* L., *Astragalus laxmannii* Jacq., *Trifolium repens* L., and *Onobrychis viciifolia* Scop. Most of these plant species (except for *Ambrosia artemisiifolia* L.) are commonly found in natural grasslands in northern China. Seeds of these plant species were obtained from the Biointeraction and Biosecurity Lab, College of Life Science, Henan University, and local commercial suppliers (see **Supplementary Table S1** for the details on these plant species). As a result, a total of 192 pots were prepared.

The experiment was conducted in an open-air environment with a movable rain shelter. On 3^rd^ March 2021, we filled 2.7kg of dry soil into an experimental pot (16cm diameter at the top, 12.5cm diameter at the bottom, and 17cm in height), corresponding to a bulk density of 1.2g cm^-3^. The seeds of each plant species were sown evenly according to the planting density of 1, 2, 4, 8, 12, and 16 spots pot^-1^, with 4 seeds in each spot. The soil moisture was adjusted to 70% of the water holding capacity (WHC) before planting. The pots were weighed and watered daily to maintain the soil moisture content at 70% WHC during the experiment. After the first 1–2 weeks of growth, seedlings were thinned to two seedlings per spot. If there were less than two seedlings in a spot, seedlings were transplanted from spare pots to make up for the difference.

### 2.3 Plant and soil sampling

After 6 months of growth, plant and soil samples were collected from each pot. The plants were cut 1cm above the soil surface to measure the aboveground biomass (AGB), in order to avoid contamination of plant tissues by soil particles and to ensure consistency across all pots. The whole soil body, including roots, was carefully taken out from each pot, and root-free bulk soil was collected from the middle part of the pot. After gently shaking the roots to remove soil loosely associated with them, the soil strongly adhering to the roots was carefully brushed off and collected as rhizosphere soil (Henneron et al., 2020). The roots remaining after rhizosphere soil collection were considered as belowground biomass (BGB). The belowground plant samples were washed with tap water, and both above- and belowground samples were dried at 105 °C for 0.5 hours, and then at 70 °C until their weights became stable (2–3 days). The root:shoot biomass ratio was calculated by dividing the root weight by the shoot weight for each pot (Vennam et al., 2023). The rhizosphere and bulk soils from each pot were individually homogenized by sieving through a 2mm sieve and then divided into four subsamples: one immediately used for the analysis of soil N mineralization rate, one stored at 4 °C for the analysis of available nutrients (e.g., NH_4_^+^, NO_3_^–^), one air-dried and sieved through a 0.25mm mesh for the analysis of other chemical properties (e.g., SOC, TN, TP, OP), and one stored at -20°C for the analysis of soil extracellular enzyme activities.

### 2.4 Soil property measurement

Soil physicochemical properties were measured with standard methods as described by Kong et al. (2022a). Soil moisture was measured by oven-drying soils at 105°C for 24h. The concentrations of SOC and TN were measured using the Walkley-Black method and the Kjeldahl method, respectively. The concentration of TP was measured colorimetrically after wet digestion with sulfuric and perchloric acid, whereas the concentration of OP was measured using the Olsen method. Soil pH was measured in a soil:water extract (1:2.5) with a pH meter (Mettler Toledo, Germany).

### 2.5 Soil extracellular enzyme activities measurement

Activities of six soil extracellular enzymes for the C, N and P cycles were measured using a microplate-scale fluorometric method with highly fluorescent compounds 7-amino-4-methylcoumarin and 4-methylumbelliferone (Kong et al., 2022c). The substrate and incubation time used for each enzyme assay are shown in **Supplementary Table S2**. The C-acquiring enzymes included in the analysis were β-1,4-glucosidase (BG), 1,4-β-D-cellobiohydrolase (CBH) and β-xylosidase (BX), while the N-acquiring enzymes were β-1,4-N-acetylglucosaminidase (NAG) and L-leucine aminopeptidase (LAP). The P-acquiring enzyme was alkaline phosphatase (ALP).

### 2.6 Soil microbial necromass carbon

The concentrations of amino sugars in soil were measured using the classical gas chromatography method as described by Zhang and Amelung (1996). The amino sugars included in this analysis were glucosamine (GlcN) and muramic acid (MurA). The MurA originates only from the bacterial cell wall and is used as a specific biomarker for bacterial necromass C, whereas GlcN is derived from both fungal and bacterial cell walls (Joergensen, 2018). Therefore, fungal (FNC) and bacterial necromass C (BNC) concentrations in the soil were quantified based on GlcN and MurA concentrations, respectively. They were calculated using Equations 1, 2 (Liang et al., 2019):


(1) $$FNC=(\frac{\text{GlcN}}{179.17}-2\times \frac{\text{MurA}}{251.23})\times 179.17\times 9$$

(2) BNC=MurA×45

where 179.17 and 251.23 are the molecular weights of GlcN and MurA, respectively, and 9 and 45 are the conversion factors of GlcN to FNC and MurA to BNC, respectively (Joergensen, 2018).

### 2.7 Soil nitrogen mineralization measurement

Soil samples (16g) were placed in a 100 mL polyethylene bottle and watered to reach the 70% WHC. The bottles were then tightly capped and incubated at 28°C for 28 days. During the incubation, the bottles were opened for 1h every 4 days to exchange air and adjust the soil moisture content to 70% WHC. After 28-day incubation, soil mineral N was measured. The concentrations of mineral N in soils before incubation were also measured. Soil NO_3_^–^ and NH_4_^+^ were extracted by mixing the soil (16g) with 80 mL of 2mol L^-1^ KCl and measured using an Autoanalyzer-AA3 (SEAL Analytical, Norderstedt, Germany). Net N ammonification rate (Ra), nitrification rate (Rn), and mineralization rate (Rm) were calculated using Equations 3–5, respectively (Kong et al., 2022a):


(3) $$Ra=(NH_{4\;i+1}^{\;+}-NH_{4\;i}^{\;+})/T$$

(4) $$Rn=(NO_{3\;i+1}^{\;-}-NO_{3\;i}^{\;-})/T$$

(5) Rm=Ra+Rn

where NH_4_^+^_i_ and NO_3_^−^_i_ are the concentrations of NH_4_^+^ and NO_3_^−^ before incubation, respectively, NH_4_^+^_i+1_ and NO_3_^−^_i+1_ are the concentrations of NH_4_^+^ and NO_3_^−^ after incubation, respectively, and T is the days of the incubation period.

### 2.8 Statistical analysis

We evaluated soil ecosystem multifunctionality based on the standardized Z-scores of soil functions (Kong et al., 2022c). Indicators used in this calculation included SOC, TN, TP, OP, NH_4_^+^, NO_3_^–^, C:N ratio, C:P ratio, N:P ratio, soil C-, N-, and P-acquiring enzyme activities, microbial necromass C (FNC, BNC and FNC: BNC ratio) and N mineralization rates (Ra, Rn and Rm). The multifunctionality index (MFI) was calculated using the *multifunc* package (https://github.com/jebyrnes/multifunc). Z-score standardization was performed across all samples (including both bulk and rhizosphere soils) to ensure comparability among functions. All functions were weighted equally in the averaging method. We did not remove any indicators due to multicollinearity, as the MFI is a descriptive metric designed to reflect the overall functional performance of ecosystems rather than to serve as a predictive model (Wang et al., 2024). Therefore, it is not sensitive to multicollinearity.

A three-way ANOVA model was used to test the direct and interaction effects of planting density (1, 2, 4, 8, 12 and 16 spots per pot), functional group (C_3_ grasses, C_4_ grasses, forbs and legumes) and sampling location (rhizosphere and bulk soils) on SOC and soil nutrients, plant biomass, root:shoot ratio, soil extracellular enzyme activities, microbial necromass C, N mineralization rates and MFI. For separate analyses within rhizosphere and bulk soils, two-way ANOVA models were used to assess the effects of planting density, functional group, and the interaction between these two factors on the same set of variables. Simple linear or logarithmic fitting was used to clarify the relationships of plant and soil metrics to planting density or biomass in different functional groups. To compare the relative strength of the effects of different functional groups, 95% confidence intervals (CIs) of the coefficients were estimated. Differences among regression coefficients were considered significant when their 95% CIs did not overlap (Berthrong et al., 2012). We conducted *post hoc* comparisons of means among different functional groups in bulk or rhizosphere soils using Tukey-Kramer HSD test. Pearson’s correlation coefficient (r) was used to evaluate pairwise linear correlation among all indicators, including both plant-related and soil function metrics. Statistical analyses were performed using JMP 10.0 (SAS Institute, Cary, USA) and the software package R (version 4.3.1).

## 3 Results

### 3.1 Effects of PFGs and planting density on plant biomass and soil nutrients

**Figure 1:**
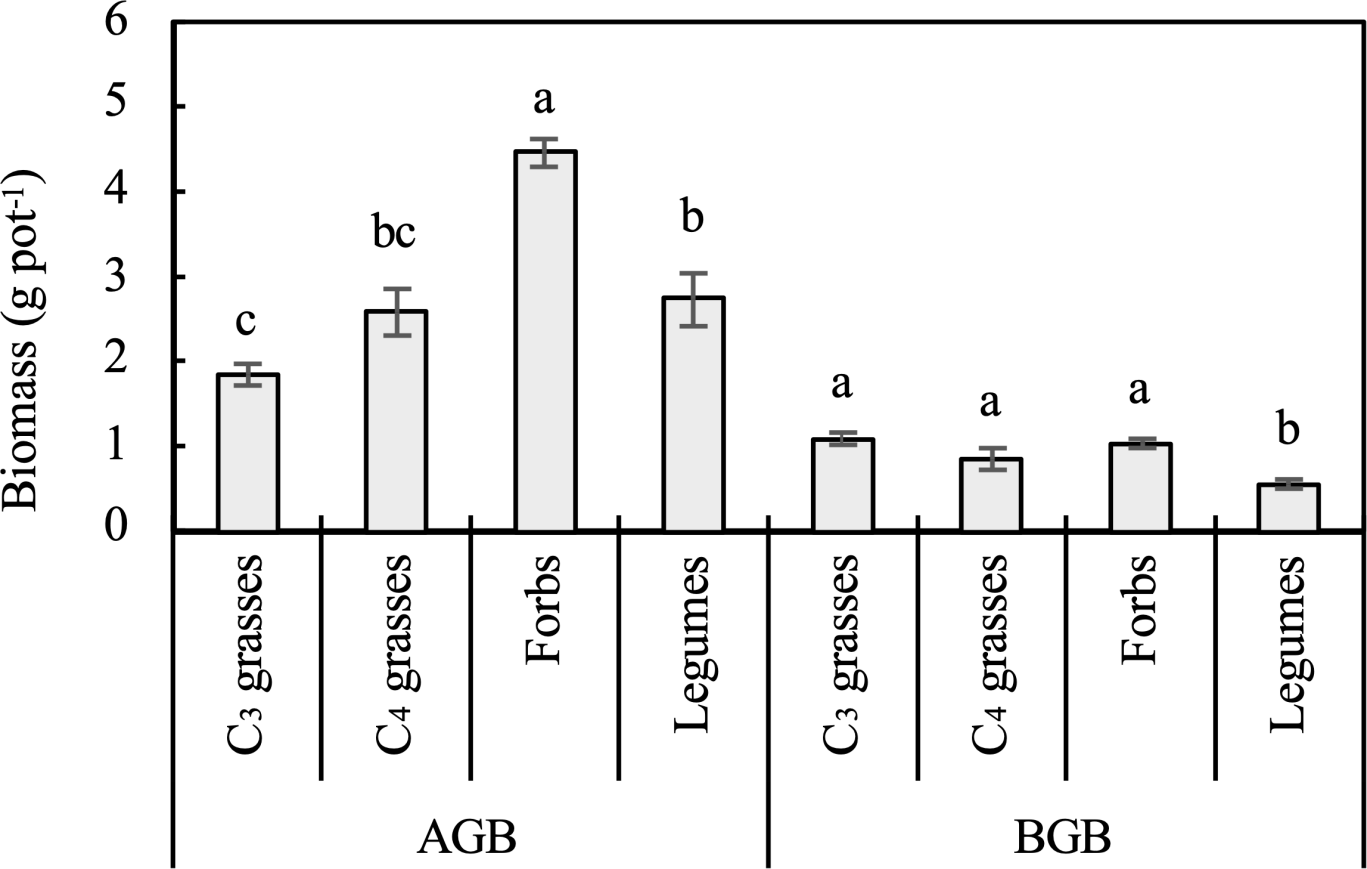
Aboveground biomass (AGB) and belowground biomass (BGB) of the four plant functional groups (PFGs) averaged across different planting densities. Lowercase letters indicate significant differences among AGB or BGB of PFGs based on the Tukey-Kramer HSD test. Error bars denote two standard errors of the mean.

The PFGs and planting density directly influenced the AGB and BGB (*P* < 0.001; **Table 1**). When averaged across all planting densities, the AGB of forbs was significantly larger than those of C_3_ grasses, C_4_ grasses, and legumes (*P* < 0.05). The BGB of C_3_ grasses, C_4_ grasses, and forbs were significantly larger than that of legumes (*P* < 0.05; **Figure 1**). The AGB and BGB tended to increase logarithmically with increasing plant density for nearly all functional groups (**Supplementary Figures S2A, B**). Among the four functional groups, legumes had the largest coefficients for the relationship between plant density and AGB (95% CI=0.097 to 1.323, *P*=0.028), but the smallest coefficient for the relationship between plant density and BGB (95% CI=0.025 to 0.237, *P*=0.019). The effect of planting density on root:shoot ratio varied with PFGs (*P* < 0.01; **Table 1**). The root:shoot ratio of legumes decreased (95% CI of coefficient = -0.075 to 0.003, *P* =0.082), while that of C_3_ grasses increased with increasing plant density (95% CI of coefficient = 0.007 to 0.175, *P*=0.038; **Supplementary Figure S2C**).

**Table 1:** The three-way ANOVA results (*P* values) for the effects of planting density (D), plant functional groups (PFGs) and location (L) on soil functions.

Effects	D	PFGs	L	D×PFGs	D×L	PFGs×L	D×PFGs×L	R^2^	RMSE	*n*
AGB	**<0.001**	**<0.001**	N/A	0.355	N/A	N/A	N/A	0.334	1.558	189
BGB	**<0.001**	**<0.001**	N/A	0.885	N/A	N/A	N/A	0.229	0.501	178
Root:shoot ratio	0.103	**<0.001**	N/A	**0.005**	N/A	N/A	N/A	0.445	0.182	178
SOC	**0.001**	**<0.001**	**<0.001**	0.588	0.692	**0.002**	0.980	0.352	0.222	366
TN	**0.011**	**<0.001**	**<0.001**	**0.017**	0.107	**<0.001**	0.073	0.575	0.028	367
TP	0.944	**<0.001**	**0.010**	0.259	**0.014**	**<0.001**	0.053	0.295	0.024	363
OP	0.574	**<0.001**	**<0.001**	0.802	0.506	0.249	0.996	0.221	0.927	362
NH_4_^+^	0.084	0.485	**<0.001**	0.153	0.919	**0.001**	0.197	0.167	0.487	347
NO_3_^–^	**0.008**	**<0.001**	**<0.001**	**0.038**	0.060	**<0.001**	0.104	0.33	1.614	346
C-acquiring enzymes	**<0.001**	**<0.001**	**<0.001**	0.247	**0.008**	**<0.001**	0.056	0.536	6.173	367
N-acquiring enzymes	**<0.001**	**0.001**	**<0.001**	0.370	**0.001**	**<0.001**	0.703	0.609	17.997	367
P-acquiring enzyme	**<0.001**	**<0.001**	0.197	0.241	0.998	**<0.001**	**0.014**	0.57	28.46	367
FNC	0.306	**<0.001**	**<0.001**	0.407	0.406	**<0.001**	0.274	0.578	0.259	342
BNC	0.370	**<0.001**	0.200	0.201	0.278	**<0.001**	0.176	0.455	0.607	366
FNC: BNC ratio	**0.014**	**<0.001**	**0.002**	0.099	**0.039**	**<0.001**	0.060	0.238	0.933	342
Ra	0.085	**<0.001**	**<0.001**	0.283	0.713	0.105	0.637	0.147	0.022	347
Rn	0.905	**<0.001**	**0.017**	**0.042**	0.901	**<0.001**	0.220	0.339	0.077	346
Rm	0.539	**<0.001**	0.263	**0.039**	0.983	**<0.001**	0.130	0.338	0.078	346
MFI	**<0.001**	**<0.001**	**<0.001**	0.091	0.499	**<0.001**	0.933	0.567	0.236	384

R^2^: coefficient of determination for the overall model. RMSE: root mean square error for the overall model. *n*: sampling size. Bold values indicate statistical significance (*P* < 0.05). N/A, data not available. AGB, aboveground biomass; BGB, belowground biomass; Root: shoot ratio, the ratio of root to shoot; SOC, soil organic carbon; TN, soil total nitrogen; TP, soil total phosphorus; OP, soil available phosphorus; NH_4_^+^, ammonium; NO_3_^–^, nitrate; FNC, fungal necromass carbon; BNC, bacterial necromass carbon; FNC: BNC ratio, the ratio of fungal necromass carbon to bacterial necromass carbon; Ra, net ammonification rate; Rn, net nitrification rate; Rm, net mineralization rate; MFI, the multifunctionality index.

**Figure 2:**
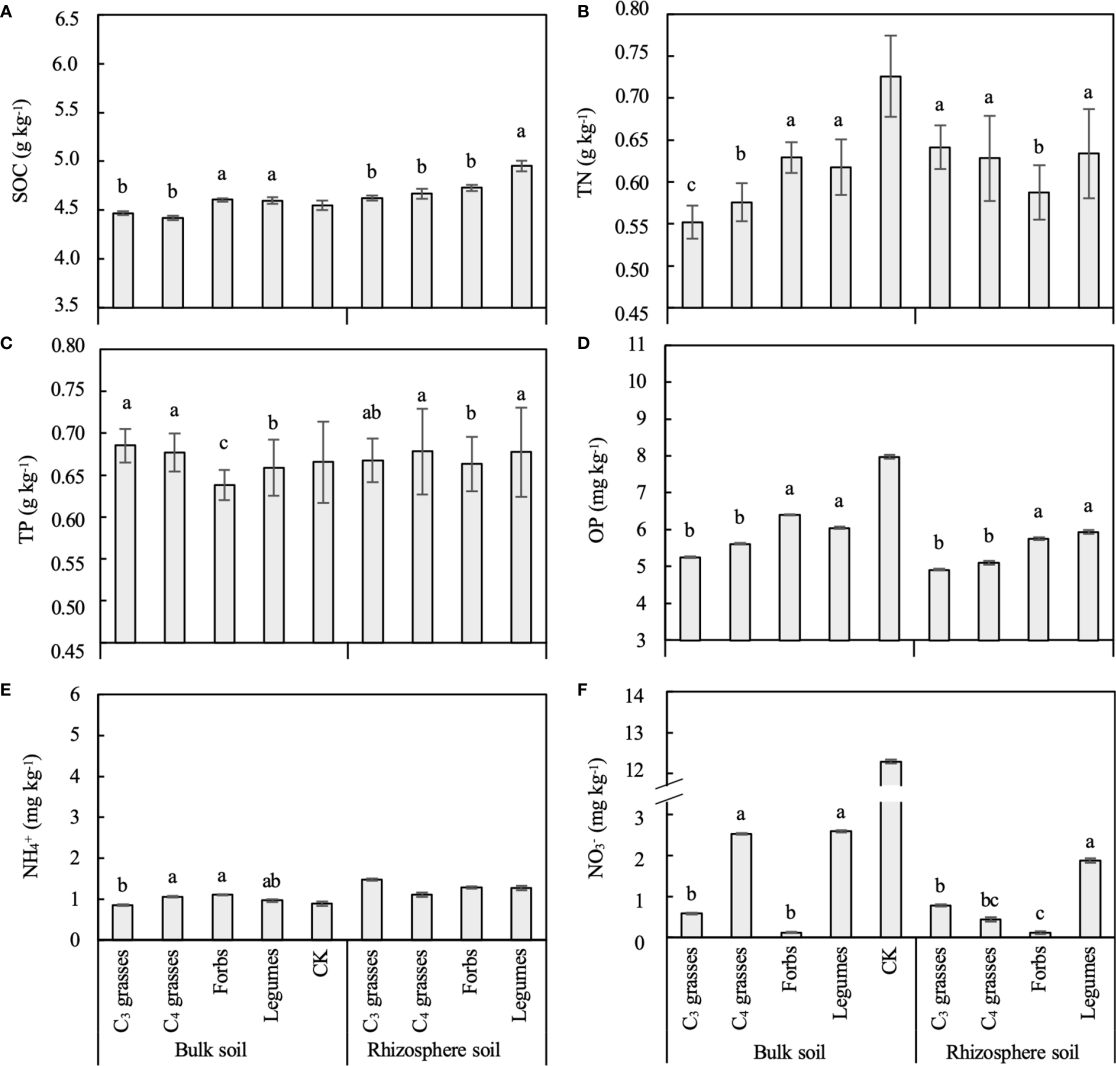
The differences between bulk and rhizosphere soils for each plant functional group (PFG) for **(A)** soil organic carbon (SOC), **(B)** total nitrogen (TN), **(C)** total phosphorus (TP), **(D)** available phosphorus (OP), **(E)** ammonium (NH_4_^+^) and **(F)** nitrate (NO_3_^–^). Lowercase letters indicate the significant differences among PFGs in bulk or rhizosphere soils based on the Tukey-Kramer HSD test. Error bars denote two standard errors of the mean. CK, control without plants (reference, not included in statistics).

The PFGs and planting density also directly influenced the concentrations of SOC and almost all soil nutrients (**Table 1**, **Supplementary Figure S3**). The SOC, OP, and NO_3_^–^ concentrations in both bulk and rhizosphere soils were significantly larger in legumes than in C_3_ grasses (*P* < 0.05; **Figures 2A, D, F**). The legumes and forbs had similar concentrations of soil OP and NH_4_^+^ (*P* > 0.05), whereas C_3_ and C_4_ grasses had similar concentrations of soil SOC, TP, and OP (*P* > 0.05; **Figures 2A, C–E**).

With increasing planting density, the concentrations of SOC, TN and NH_4_^+^ in bulk soils exhibited a logarithmic increasing trend, while the NO_3_^–^ concentration showed a decreasing trend across all functional groups, although these patterns were not all statistically significant (**Supplementary Figures S3A, C, I, K**). The TP concentration for C_3_ grasses significantly decreased with increasing planting density in bulk soils but increased in rhizosphere soils (*P* < 0.05; **Supplementary Figures S3E, F**) but this trend was not seen for the other PFGs. Moreover, changes in soil nutrients were significantly related to plant biomass. For example, TN and NO_3_^–^ were correlated to AGB, BGB and the root:shoot ratio (*P* < 0.05; **Supplementary Figure S6**).

### 3.2 Effects of PFGs and planting density on soil extracellular enzyme activities

**Figure 3:**
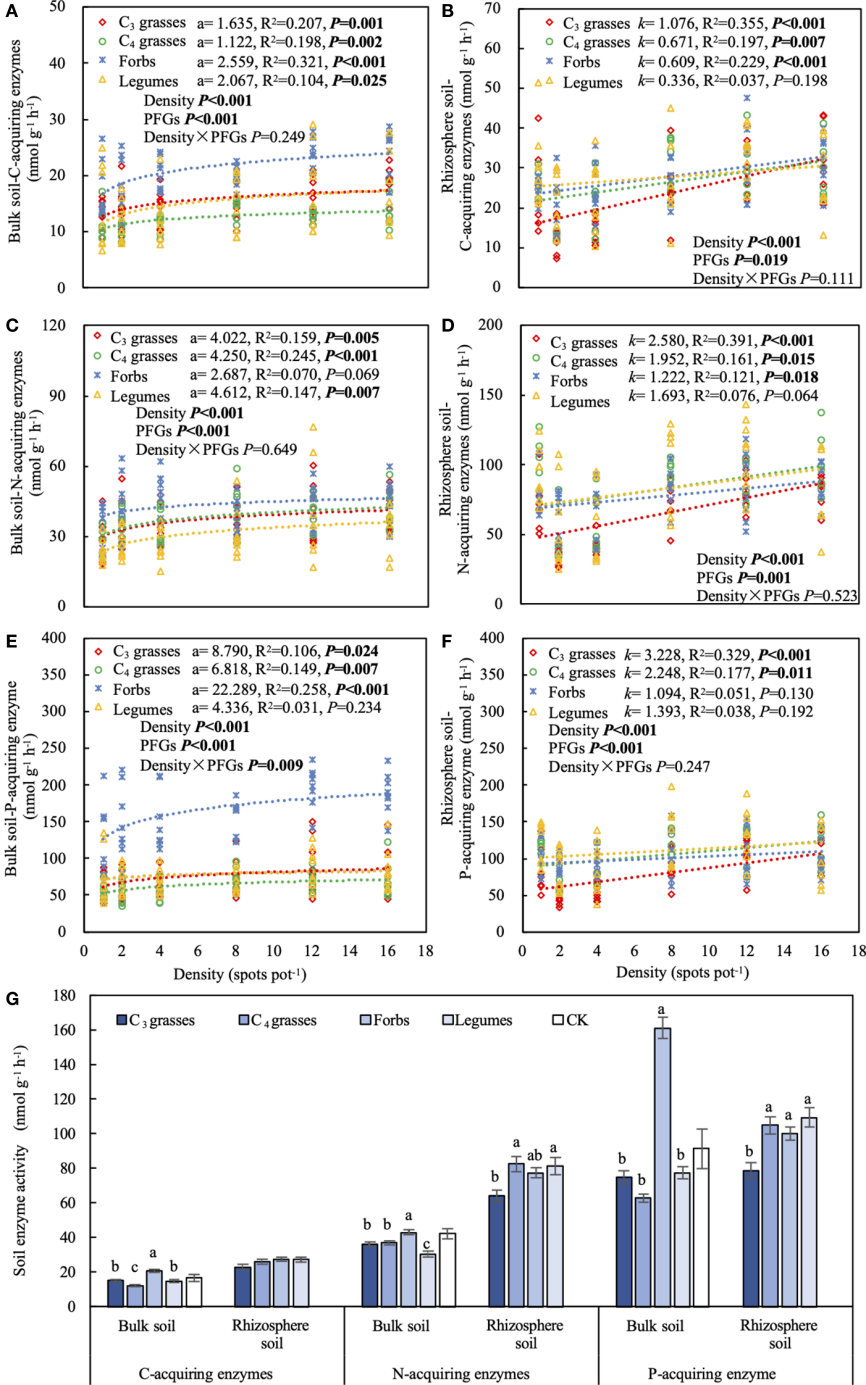
The effects of planting density on **(A)** C-acquiring enzyme activity in bulk soil, **(B)** C-acquiring enzyme activity in rhizosphere soil, **(C)** N-acquiring enzyme activity in bulk soil, **(D)** N-acquiring enzyme activity in rhizosphere soil, **(E)** P-acquiring enzyme activity in bulk soil, **(F)** P-acquiring enzyme activity in rhizosphere soil. **(G)** Activities of C-, N-, and P-acquiring enzymes for each plant functional group (PFG) in bulk and rhizosphere soils. Dashed lines indicate model fits between planting density and soil C-, N- and P-acquiring enzyme activities for each PFG. For each fit, the coefficient of the logarithmic fit (a) or slope of the linear fit (*k*), coefficient of determination (R^2^) and *P* value are shown, along with the *P* values from two-way ANOVA assessing the effects of density and PFGs on soil C-, N- and P-acquiring enzyme activities. Lowercase letters in **(G)** indicate the significant differences among the PFGs in bulk or rhizosphere soils based on the Tukey-Kramer HSD test. Error bars denote two standard errors of the mean. CK, control without plants (reference, not included in statistics).

The effects of PFGs on soil extracellular enzyme activities varied with location (*P* < 0.001; **Table 1**). When averaged across all planting densities, soil extracellular enzyme activities in bulk soils were significantly higher in forbs than the other functional groups (*P* < 0.05), whereas the activities in rhizosphere soils were higher in forbs, C_4_ grasses, and legumes than those in C_3_ grasses (**Figure 3G**). Most extracellular enzyme activities in both bulk and rhizosphere soils significantly increased with increasing planting density (*P* < 0.05; **Figures 3A-F**). For bulk soils of forbs, the coefficients relating planting density to C- and P-acquiring enzymes were the largest (95% CIs: C-acquiring enzymes = 1.5 to 3.6; P-acquiring enzyme = 11.4 to 33.2), while the coefficient for N-acquiring enzymes was the smallest (95% CI = –0.1 to 5.5; **Figures 3A, C, E**). In rhizosphere soils of C_3_ grasses, the slopes relating density to enzyme activities were the largest across enzyme types (95% CIs: C-acquiring enzymes = 0.7 to 1.5; N-acquiring enzymes = 1.6 to 3.5; P-acquiring enzyme = 1.9 to 4.6; **Figures 3B, D, F**). In addition, soil extracellular enzyme activities were significantly positively correlated with SOC and TN concentrations, but negatively correlated with the NO_3_^–^ concentration (*P* < 0.05; **Supplementary Figure S6**).

### 3.3 Effects of PFGs and planting density on microbial necromass carbon

**Figure 4:**
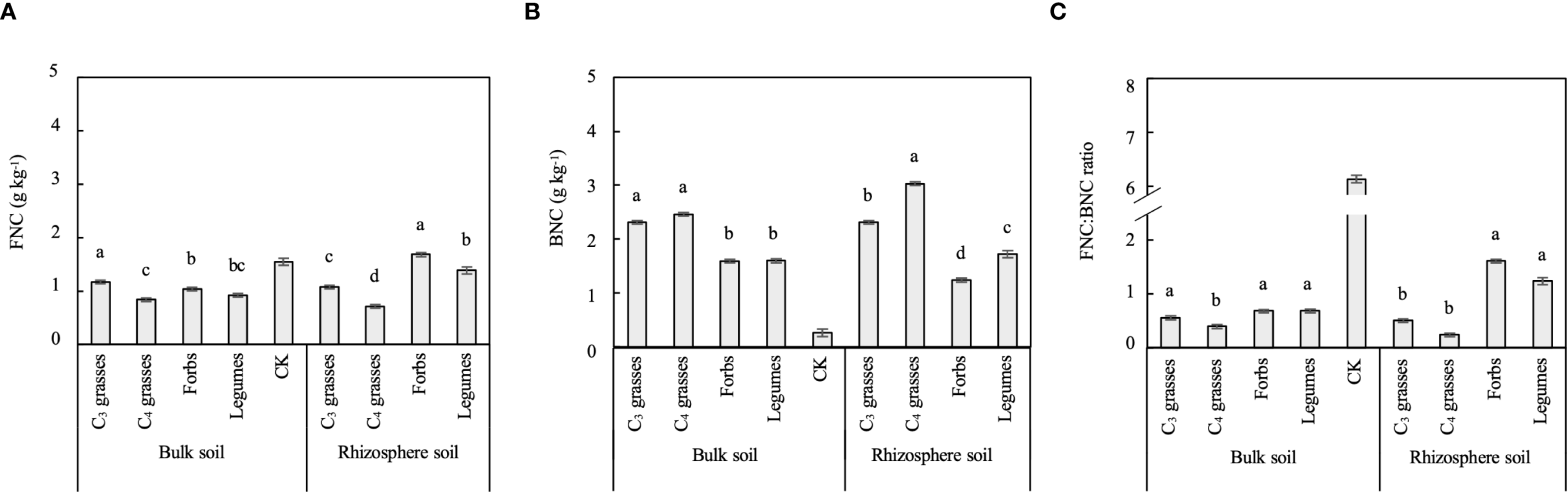
The differences between bulk and rhizosphere soils for each plant functional group (PFG) for **(A)** fungal necromass C (FNC), **(B)** bacterial necromass C (BNC) and **(C)** FNC: BNC ratio. Lowercase letters indicate the significant differences among the PFGs in bulk or rhizosphere soils based on the Tukey-Kramer HSD test. Error bars denote two standard errors of the mean. CK, control without plants (reference, not included in statistics).

Soil microbial necromass C (i.e., FNC and BNC) was significantly affected by PFGs but not by planting density, and its response to PFGs varied across locations (*P* < 0.001; **Table 1**, **Supplementary Figure S4**). The concentration of FNC in bulk soils was the highest in C_3_ grasses (1.17g kg^-1^) and the lowest in C_4_ grasses (0.83g kg^-1^), whereas that in rhizosphere soils was the highest in forbs (1.68g kg^-1^) and the lowest in C_4_ grasses (0.71g kg^-1^, *P* < 0.05; **Figure 4A**). The concentrations of BNC in both bulk and rhizosphere soils were significantly higher in C_3_ and C_4_ grasses than those in forbs and legumes (**Figure 4B**). The FNC: BNC ratios in both bulk and rhizosphere soils were significantly higher in forbs and legumes than those in C_4_ grasses (**Figure 4C**). Additionally, the microbial necromass C in rhizosphere soils of forbs and legumes were dominated by FNC, whereas those in rhizosphere soils of C_3_ and C_4_ grasses and in bulks soils from all four functional groups were dominated by BNC (FNC: BNC ratio < 1; **Figure 4C**).

### 3.4 Effects of PFGs and planting density on soil N mineralization

**Figure 5:**
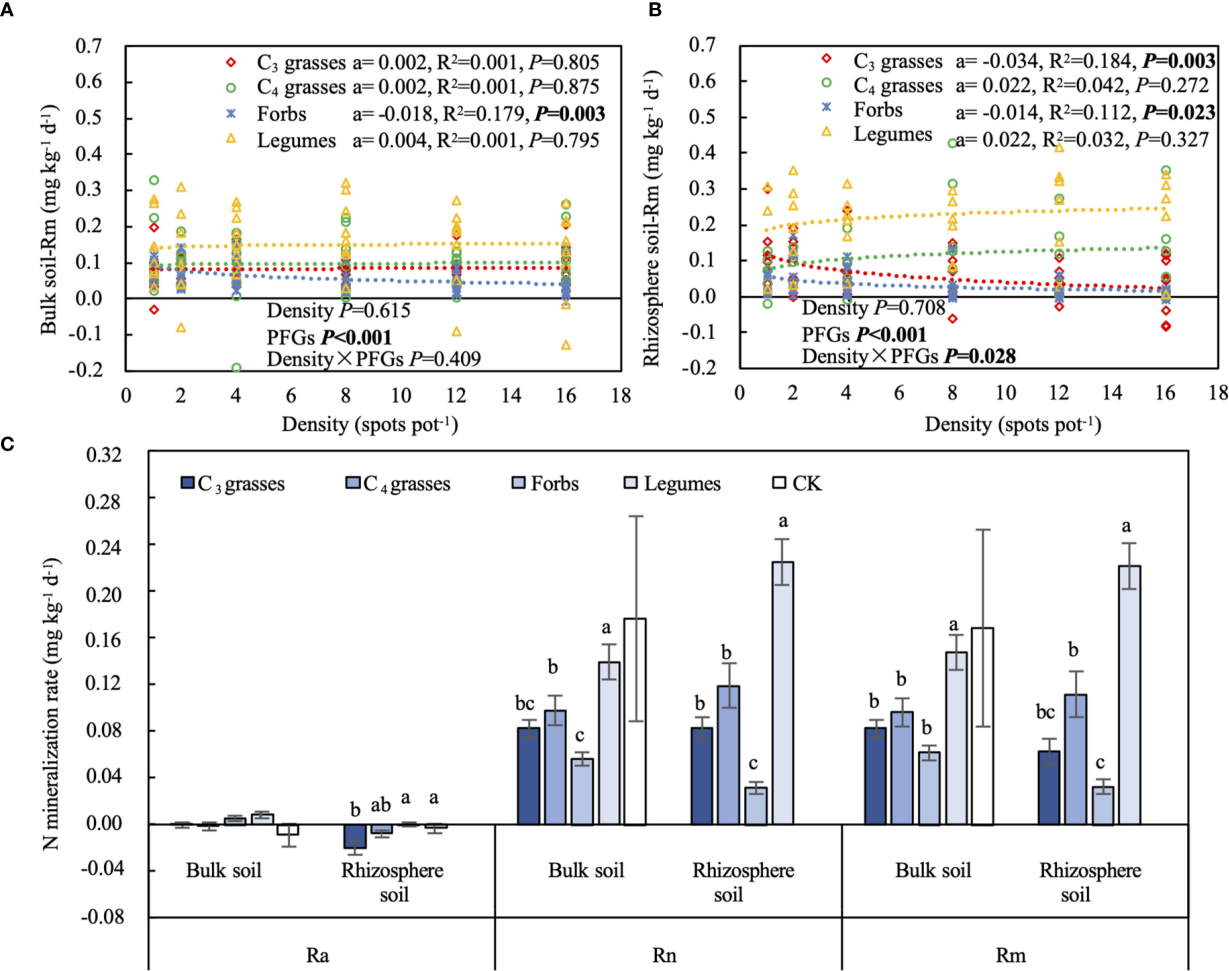
The effects of planting density on **(A)** soil net nitrogen mineralization rate (Rm) in bulk soil and **(B)** Rm in rhizosphere soil. **(C)** Soil net ammonification rate (Ra), net nitrification rate (Rn), and Rm for each plant functional group (PFG) in bulk and rhizosphere soils. Dashed lines indicate the logarithmic model fits between planting density and Rm for each PFG. For each fit, the coefficient of the logarithmic fit (a), coefficient of determination (R^2^) and *P* value are shown, along with the *P* values from two-way ANOVA assessing the effects of density and PFGs on Rm. Lowercase letters in **(C)** indicate the significant differences among the PFGs in bulk or rhizosphere soils based on the Tukey-Kramer HSD test. Error bars denote two standard errors of the mean. CK, control without plants (reference, not included in statistics).

When averaged across PFGs and planting densities, Ra was significantly higher in bulk soils than in rhizosphere soils (141% higher, *P* < 0.001). In contrast, Rn and Rm were similar between bulk and rhizosphere soils (**Figure 5C**). The Ra in bulk soils was not affected by PFGs, whereas that in rhizosphere soils was significantly higher in forbs and legumes than C_3_ grasses (*P* < 0.05; **Figure 5C**). Rn and Rm were significantly higher in legumes than other three PFGs in both bulk (42%–147% and 53%–138% higher) and rhizosphere soils (89%–605% and 98%–579% higher, *P* < 0.05; **Figure 5C**). The planting density had minimum effects on N mineralization rates (**Figures 5A, B**, **Supplementary Figure S5**). Ra did not significantly change with increasing planting density for all four PFGs (**Supplementary Figure S5A, C**). In contrast, the effects of planting density on Rn and Rm varied with PFGs (*P* < 0.05; **Table 1**). Rn and Rm decreased with increasing planting density in bulk and rhizosphere soils of forbs (95% CIs of coefficients: Rn in bulk soils = -0.029 to -0.005; Rn in rhizosphere soils = -0.024 to -0.004; Rm in bulk soils = -0.030 to -0.006; Rm in rhizosphere soils = -0.026 to -0.002; *P* < 0.05), whereas those from the other three PFGs were not affected by planting density (**Figures 5A, B**, **Supplementary Figure S5B, D**).

Ra was significantly positively correlated with the soil OP concentration but negatively correlated with TN and NH_4_^+^ concentrations, C- and N-acquiring enzyme activities and BNC (*P* < 0.05; **Supplementary Figure S6**). Rn and Rm were positively correlated with SOC and TN concentrations, but negatively correlated with P-acquiring enzyme activity (*P* < 0.05; **Supplementary Figure S6**). Additionally, the Rn and Rm in both bulk and rhizosphere soils of forbs were negatively correlated with AGB and BGB (*P* < 0.01), whereas those of legumes were positively correlated with AGB and BGB (*P* < 0.001; **Supplementary Table S3**).

### 3.5 Effects of PFGs and planting density on soil multifunctionality

**Figure 6:**
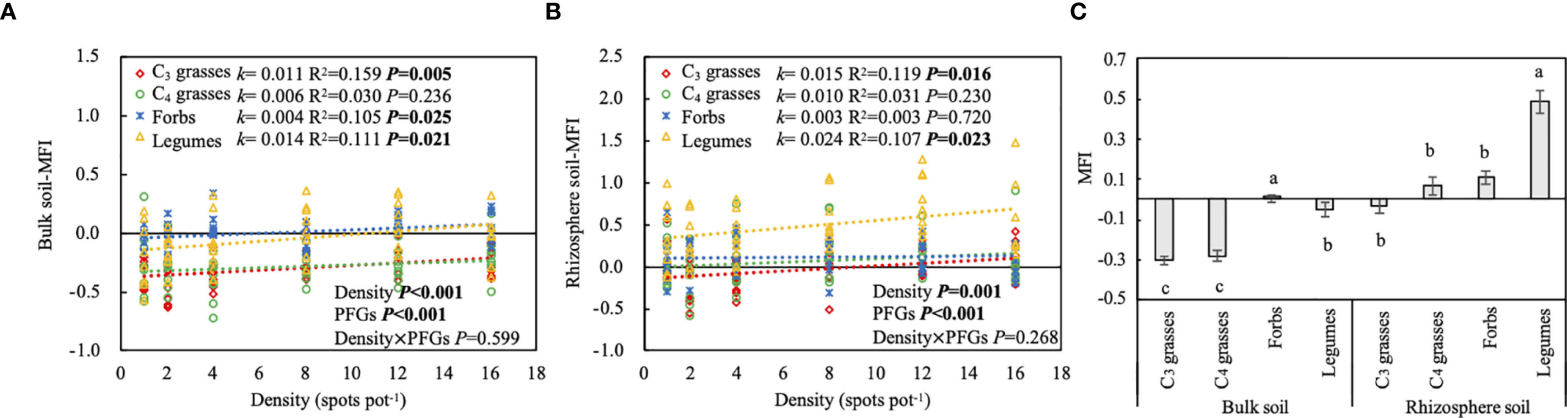
The effects of planting density on **(A)** multifunctionality index (MFI) in bulk soil, **(B)** MFI in rhizosphere soil. The differences between bulk and rhizosphere soils for each plant functional group (PFG) for MFI **(C)**. Dashed lines indicate the liner model fits between planting density and MFI for each PFG. For each fit, the slope of the linear fit (*k*), coefficient of determination (R^2^) and *P* value are shown, along with the *P* values from two-way ANOVA assessing the effects of density and PFGs on MFI. Lowercase letters in **(C)** indicate the significant differences among the PFGs in bulk or rhizosphere soils based on the Tukey-Kramer HSD test. Error bars denote two standard errors of the mean.

The effect of PFGs on the MFI varied with location (*P* < 0.001; **Table 1**). When averaged across all planting densities, the MFI in bulk soils was significantly higher in forbs than the other PFGs, whereas that in rhizosphere soils was significantly higher in legumes than the other PFGs (*P* < 0.05; **Figure 6C**). Additionally, the MFI increased linearly with planting density in both bulk and rhizosphere soils for almost all PFGs (**Figures 6A, B**). Among the four PFGs, legumes and forbs had the largest and the smallest slopes (95% CIs: legumes in bulk soils = 0.003 to 0.019; legumes in rhizosphere soils = 0.003 to 0.027; forbs in bulk soils = -0.002 to 0.010; forbs in rhizosphere soils = -0.010 to 0.014), respectively, for the relationship between density and the MFI.

## 4 Discussion

Our study showed that legumes significantly enhanced N mineralization rates and soil multifunctionality in rhizosphere soils (**Figure 5C**, **Figure 6C**). Compared to other PFGs, legumes exhibited the greatest increase in soil MFI and AGB, but the smallest increase in BGB with increasing planting density (**Figures 6A, B**; **Supplementary Figures S2A, B**). These results partially support H1 that legumes exert a more pronounced positive effect on soil functions than other PFGs, and also support H3 that the effects of planting density on soil functions vary significantly among PFGs. Additionally, soil nutrient contents and N mineralization rates logarithmically changed, either increased or decreased, with increasing density (**Supplementary Figures S3**, **S5**; **Figures 5A, B**), while extracellular enzyme activities in rhizosphere soils, microbial necromass C, and soil multifunctionality in both bulk and rhizosphere soils linear changed with density (**Figures 3B, D, F**; **Supplementary Figure S4**; **Figures 6A, B**). These mixed response patterns provide partial support for H2, which proposed nonlinear relationships between planting density and soil functions.

### 4.1 Effects of PFGs on plant growth performance and soil functions

Among the PFGs, forbs exhibited the highest AGB (**Figure 1**), likely due to their adaptive advantages (Jongejans et al., 2006). Forbs generally have a higher N uptake capacity than grasses, enabling them to allocate more biomass to leaves and improve their competitiveness for light (Zhang et al., 2020b). In contrast, grasses allocate more biomass to roots to compensate for their lower root protein content and N uptake efficiency, thereby improving their ability to explore soil resources (Poorter et al., 2015; Rehling et al., 2021). Their limited mycorrhizal associations may further necessitate this direct root investment (Van der Heijden et al., 2015). Additionally, forbs tend to be stronger competitors for light than legumes when growth is not limited by nutrients (Chen et al., 2020), which may explain our results that forbs had higher AGB than legumes. Bulk soils of forbs also showed higher extracellular enzyme activities compared to those of other PFGs. This may be associated with the greater AGB of forbs (**Supplementary Figure S6**). Higher plant productivity typically leads to greater SOC inputs into the soil, thereby enhancing soil enzyme activities and microbial biomass C (Zhang et al., 2021).

In our study, legumes had higher concentrations of SOC, OP and NO_3_^–^ than C_3_ grasses in both bulk and rhizosphere soils. This pattern is most likely attributable to the greater rhizodeposition and AGB of legumes, which not only provide C for microbes but also promote microbial decomposition of SOM and nutrient mobilization (Fornara et al., 2011; Henneron et al., 2020; Ma et al., 2022). Additionally, the relatively low BGB of legumes compared to other PFGs may have limited their ability to take up soil nutrients (**Figure 1**). Furthermore, our results also suggested that legumes exhibited higher Rn and Rm than the other PFGs in both bulk and rhizosphere soils, consistent with previous studies (Wei et al., 2019; Ripoche et al., 2021; Kong et al., 2022b; Gou et al., 2023). This result is likely attributed to the legacy effects of rhizodeposition that stimulate microbial mineralization processes (Fox et al., 2019; Gou et al., 2023; Nannipieri et al., 2023). Therefore, the higher soil nutrient contents and elevated N mineralization rates in the rhizosphere soils of legumes likely contribute to their enhanced soil multifunctionality compared to other PFGs in our study. This can be explained by the fact that increased mineralization converts organic N into inorganic forms that are more readily assimilated by soil microorganisms and plants, thereby promoting microbial activity, plant nutrient uptake, and overall soil biochemical processes such as organic matter decomposition (Van der Heijden et al., 2008; Kuzyakov and Xu, 2013). Collectively, these processes improve multiple soil functions simultaneously, leading to greater soil multifunctionality in legume rhizospheres.

In this study, soil microbial necromass C was primarily composed of BNC. This is probably due to the substantial input of labile C from plant litter and root exudates, which stimulates rapid bacterial growth and turnover (Yang et al., 2022a). The resulting BNC can be stabilized by interacting with soil microaggregates and clay minerals and become resistant to decomposition (Yang et al., 2022b). In contrast, the rhizosphere soils of forbs and legumes exhibited significantly larger FNC and FNC: BNC ratios than those of C_3_ and C_4_ grasses. This is likely due to their taproot systems, which facilitate arbuscular mycorrhizal fungi colonization (Yang et al., 2015; Hannula et al., 2019). Meanwhile, the relatively larger BNC in the soils of C_3_ and C_4_ grasses may be attributed to the rhizospheric structures and rhizosheaths, which create more favorable ecological niches for bacterial colonization (Jiang et al., 2023; Mo et al., 2023).

### 4.2 Effect of planting density on plant growth performance and soil functions

In this study, AGB and BGB increased logarithmically with planting density and reached near plateau levels when planting density reached four planting spots per pot. Among different PFGs, legumes exhibited the largest and smallest regression coefficients of AGB and BGB, respectively, with planting density. This indicates that legumes were less affected by nutrient competition but more influenced by light resources with increasing planting density. Previous studies have shown that the plant root:shoot ratio generally decreases under high planting density due to an increased stem fraction (Poorter et al., 2011; Postma et al., 2020). However, our results showed that the response of root:shoot ratio to planting density varied among PFGs. For instance, the root:shoot ratio of C_3_ grasses increased with increasing planting density, most likely due to their reliance on enhanced belowground biomass allocation to meet nutrient uptake demands. In contrast to C_3_ grasses, several factors contribute to alleviating soil nutrient limitations and increasing aboveground biomass allocation in other PFGs. These include the photosynthetic pathways of C_4_ grasses (Pearcy and Ehleringer, 1984; Sun et al., 2022), the reproductive characteristics and adaptive responses of forbs, particularly those of Asteraceae species (Jongejans et al., 2006), and the symbiotic N-fixing relationship with rhizobia in legumes (Lau et al., 2012). Together, these traits help stabilize or decrease root:shoot ratios with increasing planting density.

Our results indicated that soil extracellular enzyme activities increased with planting density in both rhizosphere and bulk soils. This is supported by a previous study showing that root biomass and the amount of root exudates increase with higher planting density, providing substrates for rhizospheric and soil microorganisms (Liu et al., 2022). Additionally, increasing planting density can intensify resource competition among individual plants, which may trigger a greater release of plant extracellular enzymes to facilitate the uptake of limited soil nutrients (Cui et al., 2019). In this study, the bulk soils of forbs had the largest coefficients for the relationships between planting density and C- and P-acquiring enzyme activities, but the smallest coefficient for the relationship with N-acquiring enzyme activity. This indicates that, as planting density increases, soil microorganisms associated with forbs may have a stronger dependence on C and P nutrients. The small coefficient for the relationship between planting density and N-acquiring enzyme activity in forb soils may be due to a resource-conservative strategy of plants as soil nutrients become limited with increasing planting density (Kato-Noguchi and Kurniadie, 2024). This strategy is characterized by high C and low nutrient demand, which can reduce N competition between plants and soil microorganisms (González et al., 2010). Moreover, forbs may release allelopathic compounds through their root exudates, potentially reducing the abundance of nitrifying bacteria, altering microbial community structure, and negatively affecting the soil N cycle (Afzal et al., 2023). For instance, a separate study by Zhang et al. (2023) demonstrated that *Ambrosia artemisiifolia* L., a forb species, can suppress soil nitrification by modulating the abundance of specific microbes (e.g., reducing the abundance of *Candidatus Nitrososphaera*). This may partly explain why the nitrification and mineralization rates of forb soils decreased with increasing planting density and biomass in both rhizosphere and bulk soils. However, these mechanisms remain speculative and require further experimental confirmation. Finally, we found that increasing planting density had a positive effect on soil multifunctionality, with legumes having the largest effect among the PFGs tested. This may be attributed to the positive correlation between density-mediated biomass increase and soil N mineralization and nitrification (**Supplementary Table S3**).

Notably, the responses of plant biomass, soil extracellular enzyme activities, microbial necromass C, and soil multifunctionality to increasing planting density did not significantly vary among PFGs. This suggests that the effects of planting density on these soil functions were consistent across PFGs, possibly due to similar plant-mediated resource inputs under higher density (Steinauer et al., 2020). It is also likely that microbial processes were more sensitive to overall carbon and nutrient inputs rather than the identity of the PFG.

## 5 Conclusions

Collectively, our results indicated that planting density and PFGs directly affect soil functions and that the effects of PFGs were greater than those of planting density. Given that previous research has primarily focused on the impacts of plant species and functional group richness on ecosystem functions, our study provides new insights into the mechanisms of how plant community change affects ecosystem functions at the functional group level. Although species identity was not explicitly modeled, the inclusion of multiple representative species per functional group strengthens the generalizability of our findings. This study was conducted in pots over a single growing season using subsoil to minimize surface legacy effects and provide a controlled environment for isolating treatment effects. While such a design offers clarity in interpreting mechanisms, it may also constrain root development, microbial complexity, and hydrological interactions. Future research should extend these findings to field-relevant conditions using surface soils and incorporate species-level variation within functional groups to better understand long-term dynamics and ecological implications.

## Data Availability

The raw data supporting the conclusions of this article will be made available by the authors, without undue reservation.

## References

[B1] Afzal M. R. Naz M. Ashraf W. Du D. L. (2023). The legacy of plant invasion: Impacts on soil nitrification and management implications. Plants 12, 2980. doi: 10.3390/plants12162980, PMID: 37631191 PMC10458916

[B2] Berthrong S. T. Pineiro G. Jobbagy E. G. Jackson R. B. (2012). Soil C and N changes with afforestation of grasslands across gradients of precipitation and plantation age. Ecol. Appl. 22, 76–86. doi: 10.1890/10-2210.1, PMID: 22471076

[B3] Calbi M. Boenisch G. Boulangeat I. Bunker D. Catford J. A. Changenet A.. (2024). A novel framework to generate plant functional groups for ecological modelling. Ecol. Indic. 166, 1122370. doi: 10.1016/j.ecolind.2024.112370

[B4] Chapman S. K. Langley J. A. Hart S. C. Koch G. W. (2005). Plants actively control nitrogen cycling: uncorking the microbial bottleneck. New Phytol. 169, 27–34. doi: 10.1111/j.1469-8137.2005.01571.x, PMID: 16390416

[B5] Chen Z. F. Xiong P. F. Zhou J. J. Yang Q. Wang Z. Xu B. C. (2020). Grassland productivity and diversity changes in responses to N and P addition depend primarily on tall clonal and annual species in semiarid Loess Plateau. Ecol. Eng. 145, 105727. doi: 10.1016/j.ecoleng.2020.105727

[B6] Cui Y. X. Bing H. J. Fang L. C. Jiang M. Shen G. T. Yu J. L.. (2019). Extracellular enzyme stoichiometry reveals the C and phosphorus limitations of microbial metabolisms in the rhizosphere and bulk soils in alpine ecosystems. Plant Soil 458, 7–20. doi: 10.1007/s11104-019-04159-x

[B7] Dai Y. L. Liao Z. Q. Lai Z. L. Bai Z. T. Zhang F. C. Li Z. J.. (2023). Interactive effects of planting pattern, supplementary irrigation and planting density on grain yield, water-nitrogen use efficiency and economic benefit of winter wheat in a semi-humid but drought-prone region of northwest China. Agric. Water Manage. 287, 108438. doi: 10.1016/j.agwat.2023.108438

[B8] Du Z. Yang L. Zhang D. Cui T. He X. Xiao T.. (2024). Optimizing maize planting density based on soil organic matter to achieve synergistic improvements of yield, economic benefits, and resource use efficiency. Sci. Total Environ. 906, 167597. doi: 10.1016/j.scitotenv.2023.167597, PMID: 37802336

[B9] FAO ITPS (2015). Status of the World’s Soil Resources-Main Report (Rome, Italy: Food and Agriculture Organization of the United Nations and Intergovernmental Technical Panel on Soils).

[B10] Fornara D. A. Bardgett R. Steinbeiss S. Zak D. R. Gleixner G. Tilman D. (2011). Plant effects on soil N mineralization are mediated by the composition of multiple soil organic fractions. Ecol. Res. 26, 201–208. doi: 10.1007/s11284-010-0777-0

[B11] Fornara D. A. Tilman D. Hobbie S. E. (2009). Linkages between plant functional composition, fine root processes and potential soil N mineralization rates. J. Ecol. 97, 48–56. doi: 10.1111/j.1365-2745.2008.01453.x

[B12] Fox A. Suter M. Widmer F. Luscher A. (2019). Positive legacy effect of previous legume proportion in a ley on the performance of a following crop of Lolium multiflorum. Plant Soil 447, 497–506. doi: 10.1007/s11104-019-04403-4

[B13] Gan D. Y. Zeng H. Zhu B. (2021). The rhizosphere effect on soil gross nitrogen mineralization: A meta-analysis. Soil Ecol. Lett. 4, 144–154. doi: 10.1007/s42832-021-0098-y

[B14] Gao D. Wang X. Fu S. Zhao J. (2017). Legume plants enhance the resistance of soil to ecosystem disturbance. Front. Plant Sci. 8, 1295. doi: 10.3389/fpls.2017.01295, PMID: 28785277 PMC5519628

[B15] Gastine A. Scherer-Lorenzen M. Leadley P. W. (2003). No consistent effects of plant diversity on root biomass, soil biota and soil abiotic conditions in temperate grassland communities. Appl. Soil. Ecol. 24, 101–111. doi: 10.1016/S0929-1393(02)00137-3

[B16] Gilmullina A. Rumpel C. Blagodatskaya E. Klumpp K. Bertrand I. Dippold M. A.. (2023). Is plant biomass input driving soil organic matter formation processes in grassland soil under contrasting management? Sci. Total Environ. 893, 164550. doi: 10.1016/j.scitotenv.2023.164550, PMID: 37295529

[B17] González A. L. Kominoski J. S. Danger M. Ishida S. Iwai N. Rubach A. (2010). Can ecological stoichiometry help explain patterns of biological invasions. Oikos 119, 779–790. doi: 10.1111/j.1600-0706.2009.18549.x

[B18] Gou X. M. Reich P. B. Qiu L. P. Shao M. A. Wei G. H. Wang J. J. (2023). Leguminous plants significantly increase soil nitrogen cycling across global climates and ecosystem types. Glob. Change Biol. 29, 4028–4043. doi: 10.1111/gcb.16742, PMID: 37186000

[B19] Grau-Andres R. Wardle D. A. Gundale M. J. Foster C. N. Kardol P. (2020). Effects of plant functional group removal on CO2 fluxes and belowground C stocks across contrasting ecosystems. Ecology 101, e03170. doi: 10.1002/ecy.3170, PMID: 32846007 PMC7757239

[B20] Gui W. Y. Ren H. Y. Liu N. Zhang Y. J. Cobb A. B. Wilson G. W. T.. (2018). Plant functional group influences arbuscular mycorrhizal fungal abundance and hyphal contribution to soil CO2 efflux in temperate grasslands. Plant Soil 432, 157–170. doi: 10.1007/s11104-018-3789-0

[B21] Han G. D. Hao X. Y. Zhao M. L. Wang M. J. Ellert B. H. Willms W.. (2008). Effect of grazing intensity on carbon and nitrogen in soil and vegetation in a meadow steppe in Inner Mongolia. Agric. Ecosyst. Environ. 125, 21–32. doi: 10.1016/j.agee.2007.11.009

[B22] Han B. He Y. C. Chen J. Wang Y. F. Shi L. N. Lin Z. R.. (2024). Different microbial functional traits drive bulk and rhizosphere soil phosphorus mobilization in an alpine meadow after nitrogen input. Sci. Total Environ. 931, 172904. doi: 10.1016/j.scitotenv.2024.172904, PMID: 38703845

[B23] Hannula S. E. Kielak A. M. Steinauer K. Huberty M. Jongen R. De-Long J. R.. (2019). Time after time: temporal variation in the effects of grass and forb species on soil bacterial and fungal communities. mBio 10, e02635–e02619. doi: 10.1128/mBio.02635-19, PMID: 31848279 PMC6918080

[B24] Henneron L. Kardol P. Wardle D. A. Cros C. Fontaine S. (2020). Rhizosphere control of soil nitrogen cycling: a key component of plant economic strategies. New Phytol. 228, 1269–1282. doi: 10.1111/nph.16760, PMID: 32562506

[B25] Herman D. J. Johnson K. K. Jaeger C. H. Schwartz E. Firestone M. K. (2006). Root influence on nitrogen mineralization and nitrification in Avena barbata rhizosphere soil. Soil Sci. Soc Am. J. 70, 1504–1511. doi: 10.2136/sssaj2005.0113

[B26] Huo C. F. Lu J. Y. Yin L. M. Wang P. Cheng W. X. (2022). Coupled of C and nitrogen mineralization in rhizosphere soils along a temperate forest altitudinal gradient. Plant Soil 500, 197–211. doi: 10.1007/s11104-022-05611-1

[B27] Jia B. Jia L. Zhang Y. M. Mou X. M. Li X. G. (2022). Leguminous Caragana korshinskii evidently enhances microbial necromass carbon accumulation in dryland soils. Catena 215, 106342. doi: 10.1016/j.catena.2022.106342

[B28] Jiang X. S. Zhong X. M. Yu G. Zhang X. H. (2023). Different effects of taproot and fibrous root crops on pore structure and microbial network in reclaimed soil. Sci. Total Environ. 901, 165996. doi: 10.1016/j.scitotenv.2023.165996, PMID: 37536594

[B29] Joergensen R. G. (2018). Amino sugars as specific indices for fungal and bacterial residues in soil. Biol. Fertil. Soils 54, 559–568. doi: 10.1007/s00374-018-1288-3

[B30] Jongejans E. De-Kroon H. Berendse F. (2006). The interplay between shifts in biomass allocation and costs of reproduction in four grassland perennials under simulated successional change. Oecologia 147, 369–378. doi: 10.1007/s00442-005-0325-8, PMID: 16400509

[B31] Kato-Noguchi H. Kurniadie D. (2024). The invasive mechanisms of the noxious alien plant species Bidens Pilosa. Plants 13, 356. doi: 10.3390/plants13030356, PMID: 38337889 PMC10857670

[B32] Kong W. B. Wei X. R. Wu Y. H. Shao M. A. Zhang Q. Sadowsky M. J.. (2022c). Afforestation can lower microbial diversity and functionality in deep soil layers in a semiarid region. Glob. Change Biol. 28, 6086–6101. doi: 10.1111/gcb.16334, PMID: 35808859

[B33] Kong W. B. Yao Y. F. Hou L. C. Bao K. Q. Zhang L. Q. Wei X. R. (2022b). Effects of vegetation presence on soil net N mineralization are independent of landscape position and vegetation type in an eroding watershed. Agric. Ecosyst. Environ. 325, 107743. doi: 10.1016/j.agee.2021.107743

[B34] Kong W. B. Yao Y. F. Hou L. C. Wang X. Wei X. R. (2022a). Site and landscape position-dependent effects of vegetation removal on soil nitrogen mineralization across five sites on China’s Loess Plateau. Catena 215, 106336. doi: 10.1016/j.catena.2022.106336

[B35] Kuzyakov Y. Xu X. L. (2013). Competition between roots and microorganisms for nitrogen: mechanisms and ecological relevance. New Phytologist. 198, 656–669. doi: 10.1111/nph.12235, PMID: 23521345

[B36] Lau J. A. Bowling E. J. Gentry L. E. Glasser P. A. Monarch E. A. Olesen W. M.. (2012). Direct and interactive effects of light and nutrients on the legume-rhizobia mutualism. Acta Oecol. 39, 80–86. doi: 10.1016/j.actao.2012.01.004

[B37] Li J. Chen D. Yang X. Fan N. Wang Y. Zhang Z. (2024). Effects of stand density, age, and drought on the size–growth relationship in Larix principis-rupprechtii forests. Forests 15, 413. doi: 10.3390/f15030413

[B38] Liang C. Amelung W. Lehmann J. Kastner M. (2019). Quantitative assessment of microbial necromass contribution to soil organic matter. Glob. Change Biol. 25, 3578–3590. doi: 10.1111/gcb.14781, PMID: 31365780

[B39] Liu S. B. He F. K. Kuzyakov Y. Xiao H. X. Hoang D. T. T. Pu S. Y.. (2022). Nutrients in the rhizosphere: A meta-analysis of content, availability, and influencing factors. Sci. Total Environ. 826, 153908. doi: 10.1016/j.scitotenv.2022.153908, PMID: 35183641

[B40] Ma W. M. Tang S. H. Dengzeng Z. M. Zhang D. Zhang T. Ma X. L. (2022). Root exudates contribute to belowground ecosystem hotspots: A review. Front. Microbiol. 13, 937940. doi: 10.3389/fmicb.2022.937940, PMID: 36274740 PMC9581264

[B41] Mariotte P. Mehrabi Z. Bezemer T. M. De-Deyn G. B. Kulmatiski A. Drigo B.. (2018). Plant-soil feedback: Bridging natural and agricultural sciences. Trends Ecol. Evol. 33, 129–142. doi: 10.1016/j.tree.2017.11.005, PMID: 29241940

[B42] Marquard E. Weigelt A. Roscher C. Gubsch M. Lipowsky A. Schmid B. (2009). Positive biodiversity-productivity relationship due to increased plant planting density. J. Ecol. 97, 696–704. doi: 10.1111/j.1365-2745.2009.01521.x

[B43] Mitchell C. E. Reich P. B. Tilman D. Groth J. V. (2003). Effects of elevated CO2, nitrogen deposition, and decreased species diversity on foliar fungal plant disease. Glob. Change Biol. 9, 438–451. doi: 10.1046/j.1365-2486.2003.00602.x

[B44] Mo X. H. Wang M. K. Zeng H. Wang J. J. (2023). Rhizosheath: Distinct features and environmental functions. Geoderma 435, 116500. doi: 10.1016/j.geoderma.2023.116500

[B45] Nannipieri P. Hannula S. E. Pietramellara G. Schloter M. Sizmur T. Pathan S. I. (2023). Legacy effects of rhizodeposits on soil microbiomes: A perspective. Soil Biol. Biochem. 184, 109107. doi: 10.1016/j.soilbio.2023.109107

[B46] Nie Z. N. Zollinger R. P. (2012). Impact of deferred grazing and fertilizer on plant population density, ground cover and soil moisture of native pastures in steep hill country of southern Australia. Grass Forage Sci. 67, 231–242. doi: 10.1111/j.1365-2494.2011.00838.x

[B47] Oñatibia G. R. Amengual G. Boyero L. Aguiar M. R. (2020). Aridity exacerbates grazing-induced rangeland degradation: A population approach for dominant grasses. J. Appl. Ecol. 57, 1999–2009. doi: 10.1111/1365-2664.13704

[B48] Pearcy R. W. Ehleringer J. (1984). Comparative ecophysiology of C3 and C4 plants. Plant Cell Environ. 7, 1–13. doi: 10.1111/j.1365-3040.1984.tb01194.x

[B49] Poorter H. Jagodzinski A. M. Ruiz-Peinado R. Kuyah S. Luo Y. Oleksyn J.. (2015). How does biomass distribution change with size and differ among species? An analysis for 1200 plant species from five continents. New Phytol. 208, 736–749. doi: 10.1111/nph.13571, PMID: 26197869 PMC5034769

[B50] Poorter H. Niklas K. J. Reich P. B. Oleksyn J. Poot P. Mommer L. (2011). Biomass allocation to leaves, stems and roots: meta-analyses of interspecific variation and environmental control. New Phytol. 193, 30–50. doi: 10.1111/j.1469-8137.2011.03952.x, PMID: 22085245

[B51] Postma J. A. Hecht V. L. Hikosaka K. Nord E. A. Pons T. L. Poorter H. (2020). Dividing the pie: A quantitative review on plant density responses. Plant Cell Environ. 44, 1072–1094. doi: 10.1111/pce.13968, PMID: 33280135

[B52] Rehling F. Sandner T. M. Matthies D. (2021). Biomass partitioning in response to intraspecific competition depends on nutrients and species characteristics: A study of 43 plant species. J. Ecol. 109, 2219–2233. doi: 10.1111/1365-2745.13635

[B53] Ripoche A. Autfray P. Rabary B. Randriamanantsoa R. Blanchart E. Trap J.. (2021). Increasing plant diversity promotes ecosystem functions in rainfed rice based short rotations in Malagasy highlands. Agric. Ecosyst. Environ. 320, 107576. doi: 10.1016/j.agee.2021.107576

[B54] Somerville C. Youngs H. Taylor C. Davis S. C. Long S. P. (2010). Feedstocks for lignocellulosic biofuels. Science 329, 790–792. doi: 10.1126/science.1189268, PMID: 20705851

[B55] Stachova T. Fibich P. Leps J. (2013). Plant density affects measures of biodiversity effects. Plant Ecol. 6, 1–11. doi: 10.1093/jpe/rts015

[B56] Steinauer K. Heinen R. Hannula S. E. De Long J. R. Huberty M. Jongen R.. (2020). Above-belowground linkages of functionally dissimilar plant communities and soil properties in a grassland experiment. Ecosphere 9, e03246. doi: 10.1002/ecs2.3246

[B57] Sun Y. F. Wang Y. P. Yan Z. B. He L. S. Ma S. H. Feng Y. H.. (2022). Above- and belowground biomass allocation and its regulation by plant density in six common grassland species in China. J. Plant Res. 135, 41–53. doi: 10.1007/s10265-021-01353-w, PMID: 34669087

[B58] Sun Y. Zang H. D. Splettstoesser T. Kumar A. Xu X. L. Kuzyakov Y.. (2020). Plant intraspecific competition and growth stage alter C and nitrogen mineralization in the rhizosphere. Plant Cell Environ. 44, 1231–1242. doi: 10.1111/pce.13945, PMID: 33175402

[B59] Van der Heijden M. G. A. Bardgett R. D. Van Straalen N. M. (2008). The unseen majority: soil microbes as drivers of plant diversity and productivity in terrestrial ecosystems. Ecol. Letters. 11, 296–310. doi: 10.1111/j.1461-0248.2007.01139.x, PMID: 18047587

[B60] Van der Heijden M. G. Martin F. M. Selosse M. A. Sanders I. R. (2015). Mycorrhizal ecology and evolution: the past, the present, and the future. New Phytol. 205, 1406–1423. doi: 10.1111/nph.13288, PMID: 25639293

[B61] Vennam R. R. Ramamoorthy P. Poudel S. Reddy K. R. Henry W. B. Bheemanahalli R. (2023). Developing functional relationships between soil moisture content and corn early-season physiology, growth, and development. Plants 12, 2471. doi: 10.3390/plants12132471, PMID: 37447032 PMC10346487

[B62] Wang N. Cheng J. Liu Y. Xu Q. Zhu C. Ling N.. (2024). Relative importance of altitude shifts with plant and microbial diversity to soil multifunctionality in grasslands of north-western China. Plant Soil 504, 545–560. doi: 10.1007/s11104-024-06641-7

[B63] Wang Y. Z. Jiao P. Y. Guo W. Du D. J. Hu Y. L. Tan X.. (2022). Changes in bulk and rhizosphere soil microbial diversity and composition along an age gradient of Chinese Fir (Cunninghamia lanceolate) plantations in subtropical China. Front. Microbiol. 12, 777862. doi: 10.3389/fmicb.2021.777862, PMID: 35281312 PMC8904968

[B64] Wei X. R. Reich P. B. Hobbie S. E. (2019). Legumes regulate grassland soil N cycling and its response to variation in species diversity and N supply but not CO_2_ . Glob. Change Biol. 25, 2396–2409. doi: 10.1111/gcb.14636, PMID: 30932274

[B65] Yan Y. Zhang Q. Buyantuev A. Liu Q. Niu J. (2020). Plant functional β diversity is an important mediator of effects of aridity on soil multifunctionality. Sci. Total Environ. 726, 138529. doi: 10.1016/j.scitotenv.2020.138529, PMID: 32305761

[B66] Yang Y. R. Dong M. Cao Y. P. Wang J. L. Tang M. Ban Y. H. (2017). Comparisons of soil properties, enzyme activities and microbial communities in heavy metal contaminated bulk and rhizosphere soils of Robinia pseudoacacia L. in the Northern Foot of Qinling Mountain. Forests 8, 430. doi: 10.3390/f8110430

[B67] Yang Y. Dou Y. X. Wang B. R. Wang Y. Q. Liang C. An S. S.. (2022b). Increasing contribution of microbial residues to soil organic carbon in grassland restoration chronosequence. Soil Biol. Biochem. 170, 108688. doi: 10.1016/j.soilbio.2022.108688

[B68] Yang Y. Dou Y. X. Wang B. R. Xue Z. J. Wang Y. Q. An S. S.. (2022a). Deciphering factors driving soil microbial life-history strategies in restored grasslands. iMeta 2, e66. doi: 10.1002/imt2.66, PMID: 38868332 PMC10989924

[B69] Yang Y. Tilman D. Furey G. Lehman C. (2019). Soil carbon sequestration accelerated by restoration of grassland biodiversity. Nat. Commun. 10, 718. doi: 10.1038/s41467-019-08636-w, PMID: 30755614 PMC6372642

[B70] Yang H. S. Zhang Q. Dai Y. J. Liu Q. Tang J. J. Bian X. M.. (2015). Effects of acbuscular mycorrhizal fungi on plant growth depend on root system: a meta-analysis. Plant Soil 389, 361–374. doi: 10.1007/s11104-014-2370-8

[B71] Yu H. W. Shen N. Yu D. Liu C. H. (2019). Effects of temporal heterogeneity of water supply and spatial heterogeneity of soil nutrients on the growth and intraspecific competition of Bolboschoenus yagara depend on plant density. Front. Plant Sci. 9, 1987. doi: 10.3389/fpls.2018.01987, PMID: 30713544 PMC6346594

[B72] Yue H. Yue W. Jiao S. Kim H. Lee Y. H. Wei G.. (2023). Plant domestication shapes rhizosphere microbiome assembly and metabolic functions. Microbiome 11, 70. doi: 10.1186/s40168-023-01513-1, PMID: 37004105 PMC10064753

[B73] Zhang X. D. Amelung W. (1996). Gas chromatographic determination of muramic acid, glucosamine, mannosamine, and galactosamine in soils. Soil Biol. Biochem. 28, 1201–1206. doi: 10.1016/0038-0717(96)00117-4

[B74] Zhang B. Cai Y. Hu S. Chang S. X. (2021). Plant mixture effects on carbon-degrading enzymes promote soil organic carbon accumulation. Soil Biol. Biochem. 163, 108457. doi: 10.1016/j.soilbio.2021.108457

[B75] Zhang R. Degen A. A. Bai Y. F. Zhang T. Wang X. M. Zhao X. Y.. (2020b). The forb, Ajania tenuifolia, uses soil nitrogen efficiently, allowing it to be dominant over sedges and Graminae in extremely degraded grasslands: Implications for grassland restoration and development on the Tibetan Plateau. Land Degrad. Dev. 31, 1265–1276. doi: 10.1002/ldr.3555

[B76] Zhang X. Huang H. Tu K. Li R. Zhang X. Wang P.. (2024). Effects of plant community structural characteristics on carbon sequestration in urban green spaces. Sci. Rep. 14, 7382. doi: 10.1038/s41598-024-57789-2, PMID: 38548813 PMC10978906

[B77] Zhang H. Li Q. Sun W. X. Guo J. Y. Liu W. X. Zhao M. X. (2023). Microbial communities in the rhizosphere soil of Ambrosia artemisiifolia facilitate its growth. Plant Soil 492, 353–365. doi: 10.1007/s11104-023-06181-6

[B78] Zhang R. C. Tielborger K. (2020a). Planting density-dependence tips the change of plant-plant interactions under environmental stress. Nat. Commun. 11, 2532. doi: 10.1038/s41467-020-16286-6, PMID: 32439842 PMC7242385

