# Supplementary material for: Plant functional group has stronger effects on soil functions than planting density: an examination with pot experiment
Source: Front Plant Sci. 2025 Sep 22;16:1652236. doi: 10.3389/fpls.2025.1652236 (PMC12497709; doi:10.3389/fpls.2025.1652236)
Supplement: Supplementary file 1 [file Table1.docx]

**TABLE S1** Plant species used and their characteristics.

| Species | Family | Functional group |
| --- | --- | --- |
| *Leymus chinensis* (Trin. ex Bunge) Tzvelev | Poaceae | C_3_ grasses |
| *Lolium perenne* L. | Poaceae | C_3_ grasses |
| *Bromus inermis* Leyss. | Poaceae | C_3_ grasses |
| *Elymus dahuricus* Turcz. | Poaceae | C_3_ grasses |
| *Panicum virgatum* L. | Poaceae | C_4_ grasses |
| *Pennisetum alopecuroides* (L.) Spreng. | Poaceae | C_4_ grasses |
| *Setaria viridis* (L.) P. Beauv. | Poaceae | C_4_ grasses |
| *Eragrostis pilosa* (L.) P. Beauv. | Poaceae | C_4_ grasses |
| *Bidens biternata* (Lour.) Merr. & Sherff | Asteraceae | Forbs |
| *Bidens pilosa* L. | Asteraceae | Forbs |
| *Ambrosia artemisiifolia* L. | Asteraceae | Forbs |
| *Ambrosia artemisiifolia* L. | Asteraceae | Forbs |
| *Medicago sativa* L. | Fabaceae | Legumes |
| *Astragalus laxmannii* Jacq. | Fabaceae | Legumes |
| *Trifolium repens* L. | Fabaceae | Legumes |
| *Onobrychis viciifolia* Scop. | Fabaceae | Legumes |
