# Supplementary material for: Plant functional group has stronger effects on soil functions than planting density: an examination with pot experiment
Source: Front Plant Sci. 2025 Sep 22;16:1652236. doi: 10.3389/fpls.2025.1652236 (PMC12497709; doi:10.3389/fpls.2025.1652236)
Supplement: Supplementary file 2 [file Table2.docx]

**TABLE S2** The substrates and incubation times used for the soil enzyme assays used in this study.

| Enzyme | Abbreviation | Substrate | Incubation time (h) |
| --- | --- | --- | --- |
| β-1,4-glucosidase | BG | 4-Methylumbelliferyl-β-D-glucopyranoside | 2 |
| 1,4-β-D-cellobiohydrolase | CBH | 4-Methylumbelliferyl-β-D-cellobioside | 4 |
| Β-xylosidase | BX | 4-Methylumbelliferyl-β-D-xylopyranoside | 4 |
| β-1,4-N-acetylglucosaminidase | NAG | 4-Methylumbelliferyl-N-acetyl  -β-D-glucosaminide | 4 |
| L-leucineaminopeptidase | LAP | L-Leucine-7-amido-4  -methylcoumarin hydrochloride | 2 |
| Alkaline phosphatase | AP | 4-Methylumbelliferyl phosphate | 0.5 |
