# Supplementary material for: Plant functional group has stronger effects on soil functions than planting density: an examination with pot experiment
Source: Front Plant Sci. 2025 Sep 22;16:1652236. doi: 10.3389/fpls.2025.1652236 (PMC12497709; doi:10.3389/fpls.2025.1652236)
Supplement: Supplementary file 3 [file Table3.docx]

**TABLE S3** The linear regression parameters between plant biomass and soil N mineralization rates.

|  |  |  | Aboveground biomass | | | | Belowground biomass | | | |
| --- | --- | --- | --- | --- | --- | --- | --- | --- | --- | --- |
|  | Location |  | C_3_ grasses | C_4_ grasses | Forbs | Legumes | C_3_ grasses | C_4_ grasses | Forbs | Legumes |
| Ra | Bulk soil | *k* | 0.003 | 0.005 | -0.001 | 0.000 | 0.002 | 0.003 | -0.000 | -0.000 |
|  |  | R^2^ | 0.027 | 0.213 | 0.012 | <0.001 | 0.009 | 0.008 | <0.001 | <0.001 |
|  |  | *P* | 0.269 | **0.001** | 0.461 | 0.992 | 0.523 | 0.595 | 0.936 | 0.974 |
|  | Rhizosphere soil | *k* | -0.001 | -0.001 | -0.000 | 0.001 | 0.000 | -0.004 | -0.006 | -0.007 |
|  |  | R^2^ | 0.001 | 0.003 | <0.001 | 0.016 | <0.001 | 0.035 | 0.034 | 0.012 |
|  |  | *P* | 0.859 | 0.777 | 0.968 | 0.495 | 0.970 | 0.314 | 0.219 | 0.543 |
| Rn | Bulk soil | *k* | 0.033 | 0.004 | -0.021 | 0.024 | 0.043 | 0.001 | -0.049 | 0.147 |
|  |  | R^2^ | 0.265 | 0.009 | 0.328 | 0.273 | 0.236 | <0.001 | 0.227 | 0.296 |
|  |  | *P* | **<0.001** | 0.546 | **<0.001** | **<0.001** | **0.001** | 0.979 | **0.001** | **<0.001** |
|  | Rhizosphere soil | *k* | 0.024 | 0.055 | -0.019 | 0.031 | 0.019 | 0.021 | -0.047 | 0.194 |
|  |  | R^2^ | 0.067 | 0.625 | 0.237 | 0.439 | 0.023 | 0.021 | 0.235 | 0.468 |
|  |  | *P* | 0.082 | **<0.001** | **0.001** | **<0.001** | 0.313 | 0.434 | **0.001** | **<0.001** |
| Rm | Bulk soil | *k* | 0.035 | 0.009 | -0.023 | 0.024 | 0.045 | 0.003 | -0.050 | 0.147 |
|  |  | R^2^ | 0.302 | 0.042 | 0.353 | 0.266 | 0.251 | 0.001 | 0.204 | 0.290 |
|  |  | *P* | **<0.001** | 0.176 | **<0.001** | **<0.001** | **<0.001** | 0.881 | **0.002** | **<0.001** |
|  | Rhizosphere soil | *k* | 0.022 | 0.054 | -0.019 | 0.033 | 0.018 | 0.017 | -0.053 | 0.187 |
|  |  | R^2^ | 0.047 | 0.578 | 0.188 | 0.474 | 0.018 | 0.013 | 0.242 | 0.436 |
|  |  | *P* | 0.148 | **<0.001** | **0.003** | **<0.001** | 0.379 | 0.537 | **0.001** | **<0.001** |

R^2^: coefficient of determination for the linear regression. *k*: slope of the linear regression. Bold values indicate statistical significance (*P* < 0.05). Ra: net ammonification rate; Rn: net nitrification rate; Rm: net N mineralization rate.
