# Supplementary material for: Plant functional group has stronger effects on soil functions than planting density: an examination with pot experiment
Source: Front Plant Sci. 2025 Sep 22;16:1652236. doi: 10.3389/fpls.2025.1652236 (PMC12497709; doi:10.3389/fpls.2025.1652236)
Supplement: Supplementary file 4 [file Image1.pdf]

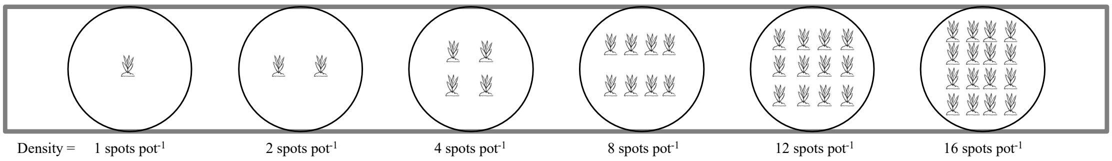

FIGURE S1 Schematic of the experimental design. All pots were monocultures, with six planting densities applied (1, 2, 4, 8, 12, and 16 spots per pot) to 16 plant species belonging to four functional groups (C_3 grasses, C_4 grasses, forbs, and legumes; four species per group). Each species × density combination was replicated twice biologically, resulting in a total of 192 pots (4 functional groups × 4 species × 6 densities × 2 replicates). Species were treated as independent experimental units within each functional group, serving as a source of biological variation to capture general patterns.
