# Supplementary material for: Plant functional group has stronger effects on soil functions than planting density: an examination with pot experiment
Source: Front Plant Sci. 2025 Sep 22;16:1652236. doi: 10.3389/fpls.2025.1652236 (PMC12497709; doi:10.3389/fpls.2025.1652236)
Supplement: Supplementary file 5 [file Image2.pdf]

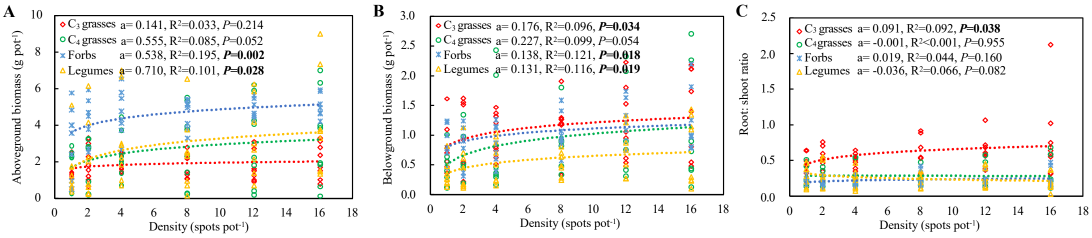

FIGURE S2 The effects of planting density on **(A)** plant aboveground biomass (AGB), **(B)** belowground biomass (BGB) and **(C)** root:shoot ratio. Dashed lines indicate logarithmic model fits between planting density and AGB, BGB or root:shoot ratio for each plant functional group. For each logarithmic fit, the coefficient of the fit (a), coefficient of determination (R^2), and *P* value are shown.
