# Supplementary material for: Plant functional group has stronger effects on soil functions than planting density: an examination with pot experiment
Source: Front Plant Sci. 2025 Sep 22;16:1652236. doi: 10.3389/fpls.2025.1652236 (PMC12497709; doi:10.3389/fpls.2025.1652236)
Supplement: Supplementary file 6 [file Image3.pdf]

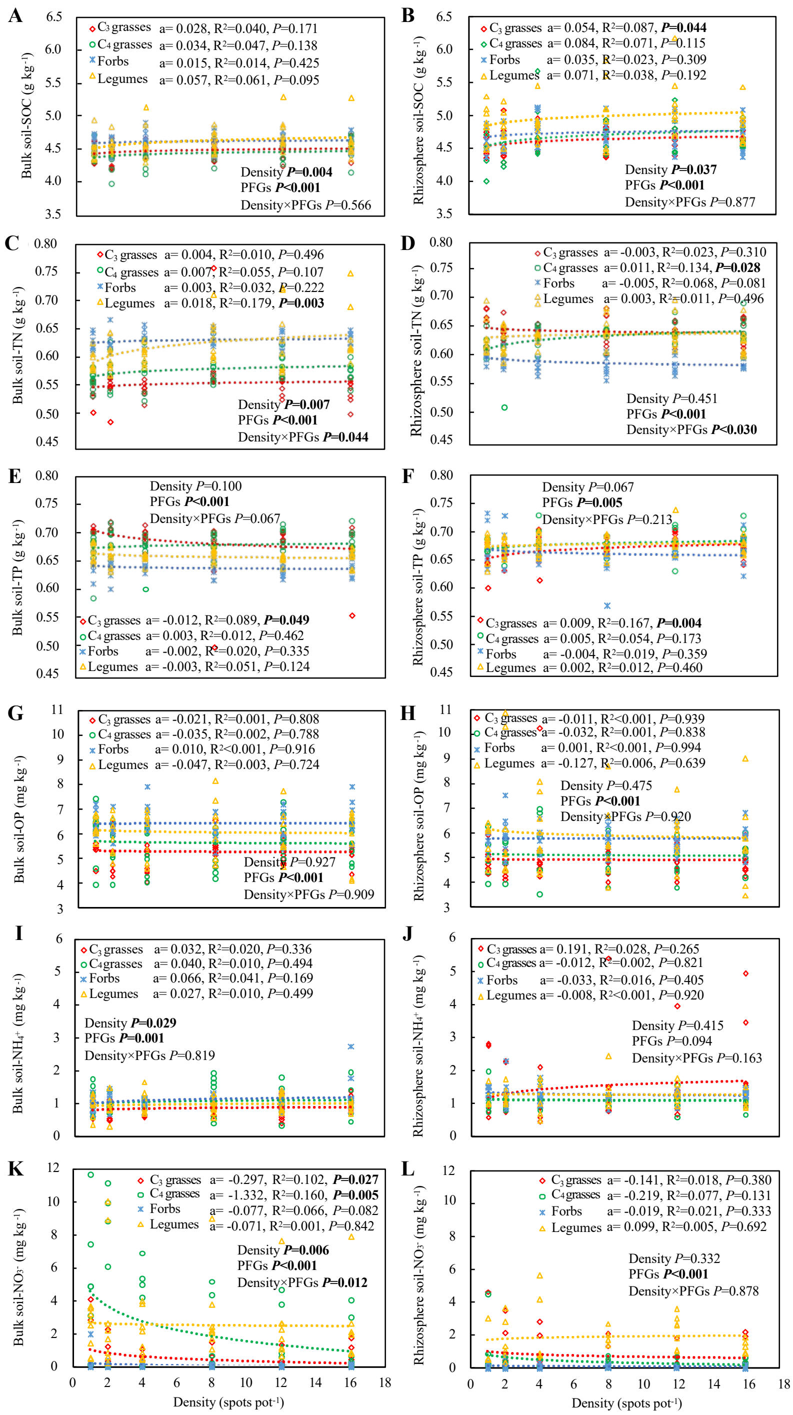

FIGURE S3 The effects of planting density on (**A**) soil organic carbon (SOC) in bulk soil, (**B**) SOC in rhizosphere soil, (**C**) total nitrogen (TN) in bulk soil, (**D**) TN in rhizosphere soil, (**E**) total phosphorus (TP) in bulk soil, (**F**) TP in rhizosphere soil, (**G**) available phosphorus (OP) in bulk soil, (**H**) OP in rhizosphere soil, (**I**) ammonium (NH_4^+) content in bulk soil, (**J**) NH_4^+ content in rhizosphere soil, (**K**) nitrate (NO_3^-) content in bulk soil, (**L**) NO_3^-content in rhizosphere soil. Dashed lines indicate the logarithmic model fits between planting density and SOC, TN, TP, OP, NH_4^+ or NO_3^- for each plant functional group (PFG). For each logarithmic fit, the coefficient of the fit (a), coefficient of determination (R^2), and *P* value are shown, along with the *P* values from two-way ANOVA assessing the effects of density and PFGs on soil nutrients.
