# Supplementary material for: Plant functional group has stronger effects on soil functions than planting density: an examination with pot experiment
Source: Front Plant Sci. 2025 Sep 22;16:1652236. doi: 10.3389/fpls.2025.1652236 (PMC12497709; doi:10.3389/fpls.2025.1652236)
Supplement: Supplementary file 7 [file Image4.pdf]

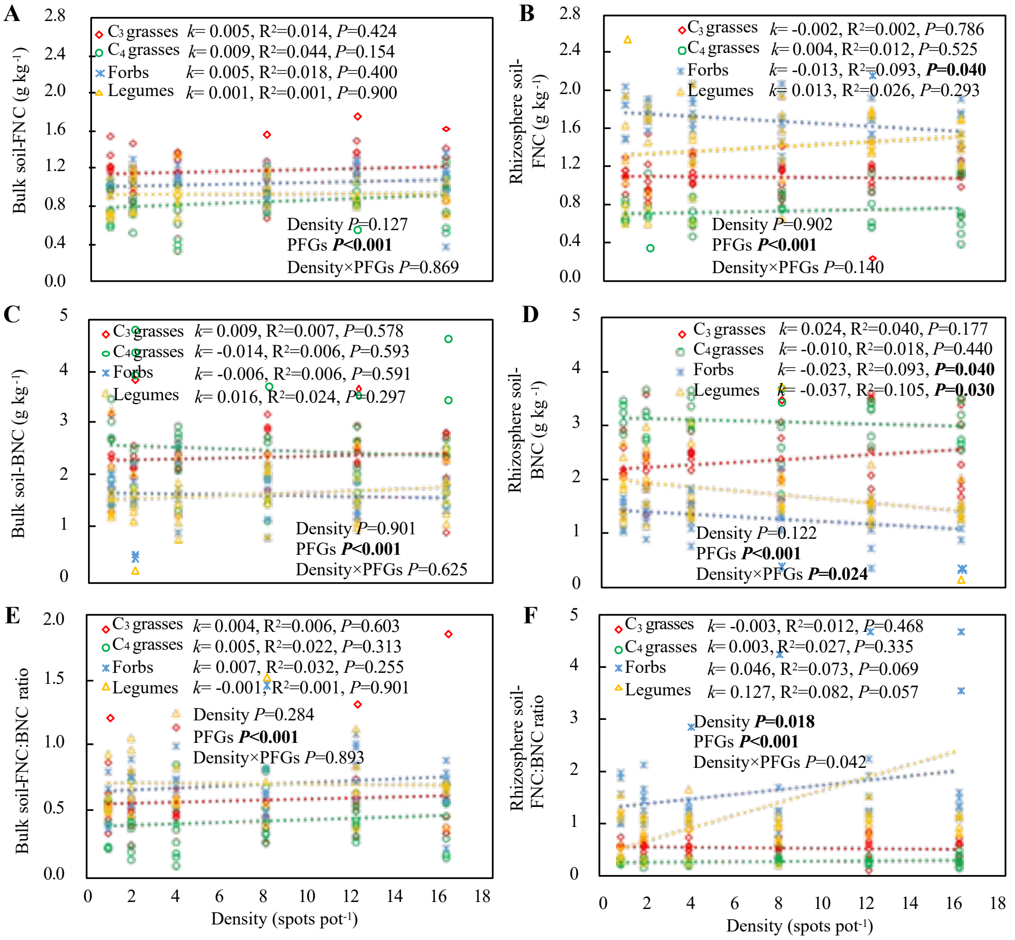

FIGURE S4 The effects of planting density on (**A**) fungal necromass C (FNC) in bulk soil, (**B**) FNC in rhizosphere soil, (**C**) bacterial necromass C (BNC) in bulk soil, (**D**) BNC in rhizosphere soil, (**E**) FNC:BNC ratio in bulk soil and (**F**) FNC:BNC ratio in rhizosphere soil. Dashed lines indicate the liner model fits between planting density and FNC, BNC or FNC:BNC ratio for each plant functional group (PFG). For each fit, the slope of the linear fit (*k*), coefficient of determination (R^2) and *P* value are shown, along with the *P* values from two-way ANOVA assessing the effects of density and PFGs on microbial necromass C.
