# Supplementary material for: Plant functional group has stronger effects on soil functions than planting density: an examination with pot experiment
Source: Front Plant Sci. 2025 Sep 22;16:1652236. doi: 10.3389/fpls.2025.1652236 (PMC12497709; doi:10.3389/fpls.2025.1652236)
Supplement: Supplementary file 8 [file Image5.pdf]

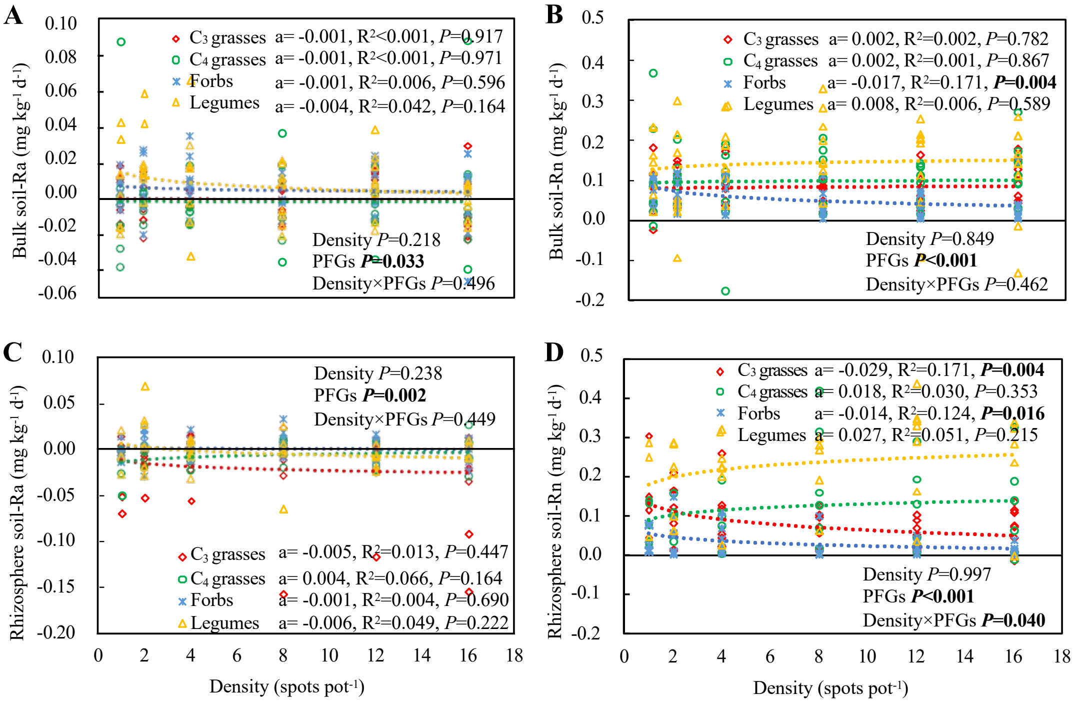

FIGURE S5 The effects of planting density on (**A**) soil net ammonification rate (Ra) in bulk soil, (**C**) Ra in rhizosphere soil, (**B**) net nitrification rate (Rn) in bulk soil and (**D**) Rn in rhizosphere soil. Dashed lines indicate the logarithmic model fits between planting density and Ra or Rn for each plant functional group (PFG). For each fit, the coefficient of the logarithmic fit (a), coefficient of determination (R^2) and *P* value are shown, along with the *P* values from two-way ANOVA assessing the effects of density and PFGs on Ra and Rn.
