# Supplementary material for: Plant functional group has stronger effects on soil functions than planting density: an examination with pot experiment
Source: Front Plant Sci. 2025 Sep 22;16:1652236. doi: 10.3389/fpls.2025.1652236 (PMC12497709; doi:10.3389/fpls.2025.1652236)
Supplement: Supplementary file 9 [file Image6.pdf]

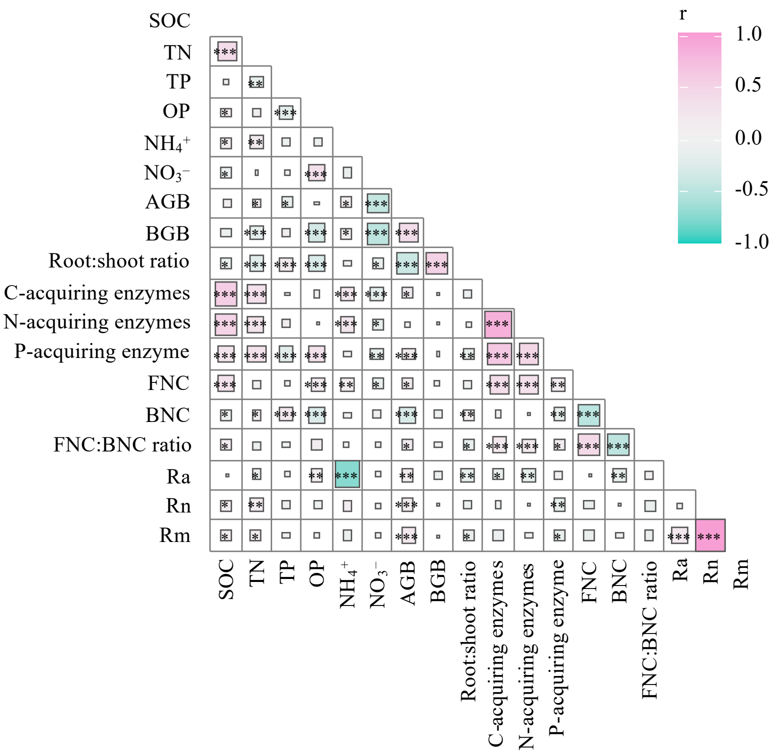

FIGURE S6 Pearson correlations among soil functional metrics. Red and green indicate positive and negative correlations, respectively. Larger squares indicate greater absolute value for the correlation coefficient. *$P < 0.05$; **$P < 0.01$; ***$P < 0.001$; SOC: soil organic carbon; TN: total nitrogen; TP: total phosphorus; OP: available phosphorus; NH_4^+: ammonium; NO_3^-: nitrate; AGB: aboveground biomass; BGB: belowground biomass; Root:shoot ratio: the ratio of root to shoot; FNC: fungal necromass carbon; BNC: bacterial necromass carbon; FNC:BNC ratio: ratio of fungal necromass carbon to bacterial necromass carbon; Ra: net ammonification rate; Rn: net nitrification rate; Rm: net N mineralization rate.
